# A premature termination codon mutation in the onion *AcCER2* gene is associated with both glossy leaves and thrip resistance

**DOI:** 10.1093/hr/uhaf006

**Published:** 2025-01-14

**Authors:** Pengzheng Lei, Meihong Pan, Shiqiang Kang, Peng Zeng, Yu Ma, Yingmei Peng, Xiushan Ma, Wei Chen, Linyu He, Haifeng Yang, Weiya Li, Shilin Zhang, Linchong Hui, Jing Cai

**Affiliations:** Vegetable Research Center, Lianyungang Academy of Agricultural Sciences, 106 Xianghaihu Road, Haizhou District, Lianyungang 222000, China; School of Ecology and Environment, Northwestern Polytechnical University, 1 Dongxiang Road, Changan District, Xi’an 710129, China; Vegetable Research Center, Lianyungang Academy of Agricultural Sciences, 106 Xianghaihu Road, Haizhou District, Lianyungang 222000, China; School of Ecology and Environment, Northwestern Polytechnical University, 1 Dongxiang Road, Changan District, Xi’an 710129, China; School of Ecology and Environment, Northwestern Polytechnical University, 1 Dongxiang Road, Changan District, Xi’an 710129, China; School of Ecology and Environment, Northwestern Polytechnical University, 1 Dongxiang Road, Changan District, Xi’an 710129, China; School of Ecology and Environment, Northwestern Polytechnical University, 1 Dongxiang Road, Changan District, Xi’an 710129, China; School of Ecology and Environment, Northwestern Polytechnical University, 1 Dongxiang Road, Changan District, Xi’an 710129, China; Vegetable Research Center, Lianyungang Academy of Agricultural Sciences, 106 Xianghaihu Road, Haizhou District, Lianyungang 222000, China; Vegetable Research Center, Lianyungang Academy of Agricultural Sciences, 106 Xianghaihu Road, Haizhou District, Lianyungang 222000, China; Vegetable Research Center, Lianyungang Academy of Agricultural Sciences, 106 Xianghaihu Road, Haizhou District, Lianyungang 222000, China; Vegetable Research Center, Lianyungang Academy of Agricultural Sciences, 106 Xianghaihu Road, Haizhou District, Lianyungang 222000, China; Vegetable Research Center, Lianyungang Academy of Agricultural Sciences, 106 Xianghaihu Road, Haizhou District, Lianyungang 222000, China; Vegetable Research Center, Lianyungang Academy of Agricultural Sciences, 106 Xianghaihu Road, Haizhou District, Lianyungang 222000, China; School of Ecology and Environment, Northwestern Polytechnical University, 1 Dongxiang Road, Changan District, Xi’an 710129, China

## Abstract

Plant epicuticular waxes (EW) play a critical role in defending against biotic and abiotic stresses. Notably, onions (*Allium cepa L.*) present a distinctive case where the mutant with defect in leaf and stalk EW showed resistance to thrips compared with the wild type with integral EW. We identified a premature stop codon mutation in the *AcCER2* gene, an ortholog of *CER2* gene in *Arabidopsis thaliana* that has been proved essential for the biosynthesis of very long-chain fatty acids (VLCFAs), in the onions with glossy leaf and stalks in our experiments. The data hinted at the possibility that this mutation might impede the elongation process of VLCFAs from C28 to C32, thereby hindering the production of 16-hentriacontanone, a primary constituent of onion EW. Transcriptomic analysis revealed substantial alterations in expression of genes in the pathways related not only to lipid synthesis and transport but also to signal transduction and cell wall modification in glossy mutants. Meanwhile, metabolomic profiling indicates a remarkable increase in flavonoid accumulation and a significant reduction in soluble sugar content in glossy mutants. These findings suggested that the enhanced resistance of glossy mutants to thrips might be a consequence of multiple physiological changes, and our integrated multiomics analysis highlighting the regulatory role of *AcCER2* in these processes. Our study has yielded valuable insights into the biosynthesis of onion EW and has provided an initial hypothesis for the mechanisms underlying thrip resistance. These findings hold significant promise for the breeding programs of thrip-resistant onion.

## Introduction

Plant cuticular waxes play an essential role in protecting plants against a wide range of stress factors, encompassing both biotic and abiotic [1]. They form a critical protective layer, safeguarding plants from various challenges such as extreme temperatures [2], drought [3], ultraviolet radiation [4], and attacks from pathogens and herbivores [5, 6]. Cuticular wax consists of two main parts, the intracuticular wax, intricately layered within the cutin matrix, and the epicuticular wax (EW), which is superimposed on the matrix as a film and crystalline structures [7, 8]. These waxes are complex organic blends, predominantly comprising very long chain fatty acids (VLCFAs) that typically range from 26 to 34 carbon atoms in length and a variety of derivatives such as alcohols, alkanes, aldehydes, ketones, wax esters, and cyclic compounds such as terpenes and sterols, contributing to their complex and multifunctional nature [9].

The biosynthesis of epidermal waxes is a complex biological process involving multiple enzymes, which could be divided into three stages. Initially, the biosynthesis of long-chain fatty acids (LCFAs) begins with acetyl-CoA, and enzymes add two-carbon units to elongate the fatty acid chain with acyl carrier protein (ACP). Once the chain reaches a certain length, typically C16 or C18, the fatty acids are further released by the action of fatty acyl-ACP thioesterases (FAT) and esterified by long-chain acyl-CoA synthetase 1 (LACS1) and 2 (LACS2) enzymes [7, 10, 11]. Subsequently, C16 and C18 acyl-CoAs serve as substrates for further elongation within the endoplasmic reticulum (ER) by the multisubunit fatty acid elongase complexes (FAE), consisting of β-ketoacyl-CoA synthase (KCS), β-ketoacyl-CoA reductase, β-hydroxy acyl-CoA dehydratase, and enoyl-CoA reductase. As the chain length extends to 28 carbons, the involvement of CER2 (ECERIFERUM2) becomes crucial to facilitate elongation to C30 or even longer carbon chains [8, 12, 13]. Finally, VLCFA-CoAs can enter either the acyl reduction pathway, producing primary alcohols, or a decarbonylation pathway, leading to the formation of aldehydes and alkanes [7, 14]. This bifurcation in the metabolic process underscores the versatility of VLCFA-CoAs in contributing to the diversity of compounds in plant cuticular waxes.

Numerous studies have shown that the presence of plant EW is essential for insect resistance, by affecting insect oviposition and phagocytosis [6, 15]. Herbivorous insects need to attach to the plant surfaces for feeding, and presence of EW can effectively weaken the attachment of insects, thus reduce insect damage [16, 17]. For instance, flea beetles (*Phyllotreta cruciferae*) showed lower feeding rates on *Brassica* species with higher leaf EW content and were mainly concentrated at the leaf margins [16]. Similarly, a study on alfalfa (*Medicago sativa* L.) found that alfalfa aphids (*Therioaphis maculata*) tend to feed older leaves with lower EW content [18]. However, intriguingly, some studies have indicated that mutants deficient in EW may exhibit enhanced resistance to insects compared to their wild-type (WT) counterparts [13, 19]. A recent study revealed a complex trade-off between the accumulation of cuticular waxes and jasmonic acid in maize. When wax synthesis is obstructed, the resultant surge in jasmonic acid levels leads to an interesting phenomenon: the maize plants deficient in EW exhibit enhanced resistance to herbivore attacks [20], which shed new light on the intricate balance between different pathways of plant defense.

Onions (*Allium cepa* L*.*) are a crop of significant economic value, cultivated and utilized worldwide [21]. The adaptability of onions to different climatic conditions and soil types contributes to their widespread cultivation and popularity [22]. The flowering stalks and leaf surfaces of onions are rich in EW, predominantly consisting of 16-hentriacontanone, accompanied by smaller quantities of 1-octacosanol and 1-triacontanol-1 [23–25]. This composition imparts a visually striking frosty-white appearance to the stalks and leaves of the onion, especially prominent during the blooming period. Onions deficient in EW visually appear with glossy leaves and stalks, and it has been established from prior research that this glossy phenotype is governed by a recessive locus [25]. Fascinatingly, previous studies have uncovered that onion accessions with reduced levels of EW exhibit enhanced resistance to onion thrips (*Thrips tabaci* L) [26, 27]. Nevertheless, the precise genetic determinants responsible for the glossy phenotype remain elusive while some quantitative trait loci (QTLs) associated with the regulation of 16-hentriacontanone and primary fatty alcohols have been pinpointed, and the intricate genetic mechanisms that confer increased resistance to thrips have not yet been thoroughly investigated.

In this study, we presented findings suggesting that the *AcCER2* gene may have a significant role in the biosynthesis of onion EW. It appeared that the mutation of *AcCER2* in glossy-type (GT) accessions may interfere with the elongation of VLCFAs from C28 to C32. Such a disruption potentially leads to a reduced presence of 16-hentriacontanone precursors, contributing to the observed defects in EW in the onion. Furthermore, transcriptomic analysis indicated that GT accessions potentially may attain thrips resistance via alterations in cell wall composition and the regulation of signal transduction. Metabolomic profiling also revealed a notable reduction in soluble sugar content within the flowering stalks of these onions, accompanied by an elevated accumulation of flavonoids. These insights are significant in enhancing our comprehension of the EW biosynthesis pathway in onions and hold considerable promise for the breeding of onion varieties with inherent resistance to thrips.

## Results

### Morphological characteristics and statistics of agronomic traits

**Figure 1:**
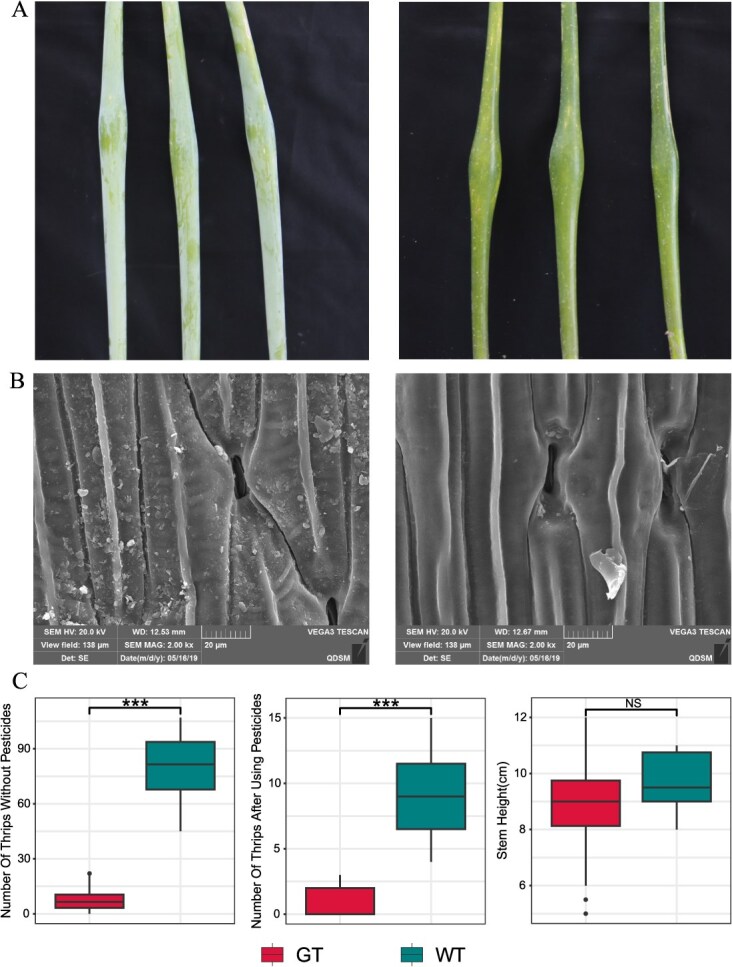
Characterization of the EW and statistics of agronomic traits in onions. (A) Flowering stalks of WT (left) and GT (right). (B) SEM of WT (left) and GT (right) leaves. Scale bars = 20 mm. (C) Boxplots for comparison of WT and GT agronomic traits. The statistical significance of the difference between the GT and WT was confirmed with a Wilcoxon test. ‘*’ means *P-*value < 0.05, ‘***’ means *P*-value < 0.01, ‘NS’ means *P*-value ≥ 0.05.

WT onion accessions used in this study, following autumn planting and germination of the seed bulbs, exhibited a frost-white layer of waxes on the surfaces of both leaves and flowering stalks (Fig. 1a). However, GT accessions did not form a visibly white waxy layer and the overall plant appeared glossy green. Although this layer was not visible to the naked eye, a thin, transparent cuticle was still perceptible when touching the flowering stalk (Fig. 1a). Observations of the ultrastructure of onion leaves have revealed that the leaves of WT accessions were densely adorned with wax crystals. (Fig. 1b). In the GT accessions, however, only a few sparse wax crystals were observed on the leaves (Fig. 1b). This suggested that GT accessions are not completely devoid of EW, instead, the scarcity of wax crystals renders them virtually undetectable to the naked eye.

EW on plants were typically associated with reduced insect adherence, contributing to protection against insect infestations [16, 17, 28]. Intriguingly, previous studies have disclosed a fascinating contrast in onions, wherein the GT accessions have shown notable resistance to thrips [27]. In line with expectations, WT accessions exhibited an adherence of 45 to 107 thrips on their leaves without the use of insecticides, whereas the GT accessions showed a maximum adherence of only 22 thrips, and even one accession (an onion inbred line named 19 234 bred by Lianyungang Academy of Agricultural Sciences) demonstrated absolute resistance (zero adherence). Following insecticide application, the number of thrips on WT accessions decreased to an average of nine while on GT accessions, it fell to three or fewer. Importantly, despite their resistance to thrips, the GT accessions did not exhibit a significant decline in performance of agronomic traits. Furthermore, there was no notable difference in the height of the flowering stalks between the two groups (Fig. 1c). Such results implied that these GT accessions have great potential as a genetic resource for developing thrip-resistant onion strains.

### The inheritance pattern and genetic mapping of GT phenotype in onions showed *AcCER2* as a key candidate gene

In order to elucidate the genetic mechanisms behind the phenotypic variations of GT accessions. We selected the onion inbred line 19 016 and its corresponding GT mutant for hybridization. The resulting F1 progeny uniformly exhibited a wax-covered phenotype. Subsequent self-pollination of the F1 generation led to a clear phenotypic segregation in the F2 generation, where the ratio of WT to GT individuals was ~3:1. This observed segregation ratio suggested that the absence of EW on the onion is a qualitative trait controlled by a single recessive locus.

To determine the causal gene mutation for the glossy phenotype in onion, we conducted bulked segregant analysis (BSA). From the F2 population, 30 WT individuals with normal EW and 30 glossy mutants with defect in EW were selected to form a pool of WT and GT, respectively. High-depth sequencing (30×) was performed on pooled DNA from these samples, followed by variant detection based on a high-quality reference genome of onion. After filtering out low-quality and missing sites, we identified a total of 144.47 million variant sites. The sites that showed a homozygous alternate (alt) state in both groups were further omitted as they are attributable to differences between the 19 016 inbred line and the reference genome (Table S1, S2). Remarkably, only 0.25% (259369) of the variants occurred in exonic regions, which is significantly lower than the proportion of exonic regions in the entire genome (0.45%; *P* < 0.01, Fisher’s exact test) (Table S3, Fig. S1). The phenotypic statistics of the F2 generation suggested that the glossy phenotype is likely caused by a recessive gene mutation. Therefore, heterozygous sites shared by both groups were filtered out in subsequent analyses.

**Figure 2:**
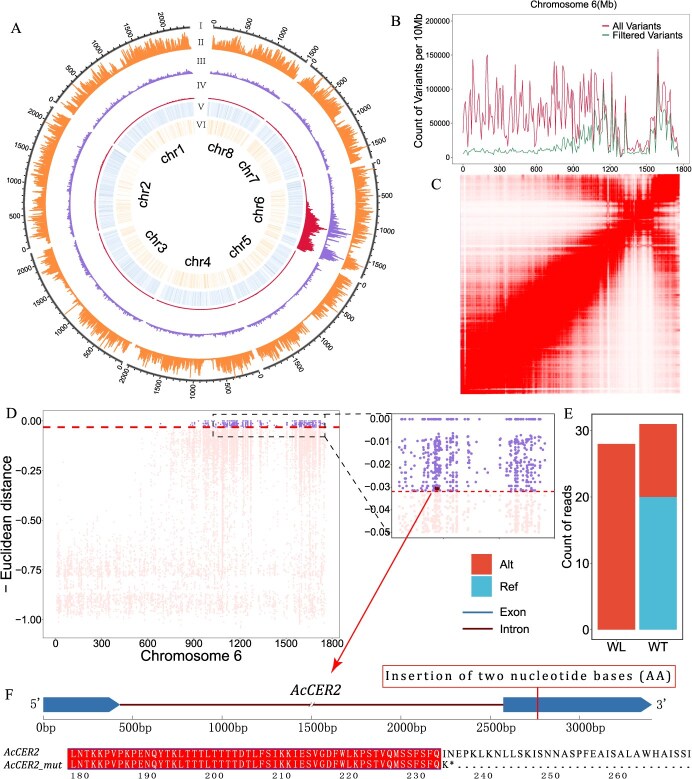
Identification of candidate loci regulating synthesis of onion EW using BSA. (A) Overview of variants in onion genome. Track I corresponds to chromosome length. Tracks II represents the distribution of all variants. Track III represents the distribution of variants after filtering out the heterozygous sites shared by both pools. Track IV represents the distribution of ED. Track V heat map represents the gene density of onion reference genome. Track VI heat map represents the TE density of onion reference genome. (B) The line plot of the distribution of variants on onion chromosome6. (C) Interaction map of Hi-C data of onion chromosome 6. The interaction intensity increases from white to red of corresponding bin pair of the chromosome. (D) The dot plot of ED between allelic frequencies and ‘ideal frequencies’ for filtered variants on chromosome 6. The dots above the dashed line represent the nearest sites (TOP 5%), and the plot on the right is a zoomed-in view of the corresponding interval. (E) The stack plot of read counts at position 1 155 655 200 on chromosome 6. (F) The gene structure of *AcCER2* and the insertion site of the mutation, and the diagram below shows the corresponding amino acid coding sequence of the *AcCER2*.

Interestingly, after filtering these sites, most genomic regions showed a significant decrease in variant density. However, an interval spanning 808 Mb on chromosome 6, from 958 to 1766 Mb, did not exhibit notable changes, suggesting that most variants in this segment were not derived from heterozygous sites in the parents (Fig. 2a and b). Then, Euclidean distance (ED) was utilized to characterize segregation distortion across the genome by calculating the distance for each variant site between WT and GT pools (DNA bulks) aimed to infer potential candidate regions exhibiting significant deviations from expected segregation ratios. To minimize the effect of variant density on our analysis, we employed a sliding window approach to calculate the average ED across variant sites for each region (total ED of variant sites within the windows/number of variant sites within the windows). The results revealed that the region from 958 to 1766 Mb on chromosome 6 displayed significantly higher ED (ED > top 99%) compared to other genomic areas (Fig. 2a). This finding was supported by additional methodologies, including the standard G-statistic, allele frequency method, and significant structural variant method, all of which corroborated our results (Fig. S2). Typically, regions near centromeres exhibit a higher degree of linkage due to physical proximity. Hi-C data alignment with the onion reference genome confirmed our hypothesis, indicating that the centromere of chromosome 6 near the chromosomal end (Fig. 2c). Strong interactions in this area were observed by heat map, suggesting a close spatial arrangement, which might impede chromosomal crossover, thereby creating a large linked region. It could be a significant challenge for the fine mapping in our subsequent analyses.

To precisely pinpoint the genetic locus responsible for glossy phenotype in onions, we narrowed our focus on variant sites within the promoter regions (2000 bases upstream of the start codon) and coding regions of genes in the candidate interval on chromosome 6. Given that the trait in question is qualitative and caused by a recessive mutation, the ideal genotype for causal mutation site would be homozygous with mutant allele in the GT group, and homozygous with WT allele and heterozygous at a ratio of one-third to two-thirds, respectively, in the WT group. So, the allele frequency ratio for mutant to WT in the WT group should be 1:2, and we referred to these genotypic frequencies as ‘ideal frequency’. A more targeted approach was used to quantify the likelihood of a site being a candidate locus by calculating the ED between the genotype frequencies at variant sites and ‘ideal frequency’, rather than characterizing segregation distortion by using ED across variant sites between WT and GT. The top 5% of sites nearest to the ‘ideal frequency’ were identified as candidate sites within the interval (Fig. 2d). Further functional impact analysis of these variant sites revealed a significant insertion of two nucleotide bases (AA) at position 1 155 655 200 on chromosome 6. This insertion led to a frameshift mutation in the gene *Acep.chr6.004871*, truncating its encoded protein from 419 to 234 amino acids and disrupting the CoA-acyltransferase domain (Fig. 2f, Fig. S3a). Although the mutant protein retained partial structural features of the CoA-acyltransferase domain, the loss of numerous conserved sites downstream of the mutation likely rendered it nonfunctional (Figs S3a and S4). Statistical analysis of the genotypic frequency at this site showed 28 reads supporting the AA insertion in the GT group, while in the WT group, only 10 reads supported the insertion, and 21 reads aligned with the reference genome sequence (Fig. 2e). This distribution aligns well with the hypothesized ideal genotypic frequency for a candidate site.

To validate the effect of the *AcCER2* gene on onion phenotypes, we selected an additional set of 10 onion accessions, including five WT and five GT onions. To minimize potential influences of closely related genetic backgrounds, these accessions were chosen to include a variety of maturity periods, bulb colors, and bulb shapes (Fig. S5). Sequencing of the *AcCER2* gene in these samples identified an insertion of two nucleotide bases (AA) unique to all GT onions (Fig. S6). Furthermore, polymorphism analysis at multiple loci within the *AcCER2* indicated that all GT onions shared the same haplotype, suggesting a single origin of this mutation (Fig. S7). Since most onion accessions in China were introduced from other regions such as the USA and Europe in the late 1980s and subsequently improved through local hybridization. we speculated that this mutation may have existed at a low genotype frequency in various onion populations prior to its introduction to China and remained in a heterozygous state. This might also provide a reasonable explanation for why a small number of imported accessions have produced GT individuals after being self-pollinated for several generations.

It was also important to highlight that the validation samples included a pair of accessions (V24-GT and V24-WT) from the same inbred line that displayed trait segregation. Sequencing of the V24-WT individual indicated that the aforementioned polymorphic sites were heterozygous (Fig. S8), whereas these sites were homozygous in the V24-GT individual. This finding further supports the hypothesis that the GT phenotype arises only when a homozygous mutation is present in the *AcCER2* gene.

### Wax composition and biosynthetic pathway comparison of GT and WT accessions confirmed the expected difference in VLCFA due to loss of function mutation in *AcCER2*

In-depth analysis identified *Acep.chr6.004871* as an acetyltransferase, homologous to *AtCER2* in *Arabidopsis thaliana*. It is known that EW are composed of VLCFAs and their derivatives, and AtCER2, along with the FAE complex, plays a crucial role in elongating C28 fatty acids to C32 fatty acids. Thus, we have named *Acep.chr6.004871* as *AcCER2*, proposing it as the potential gene responsible for the absence of EW in onions.

**Figure 3:**
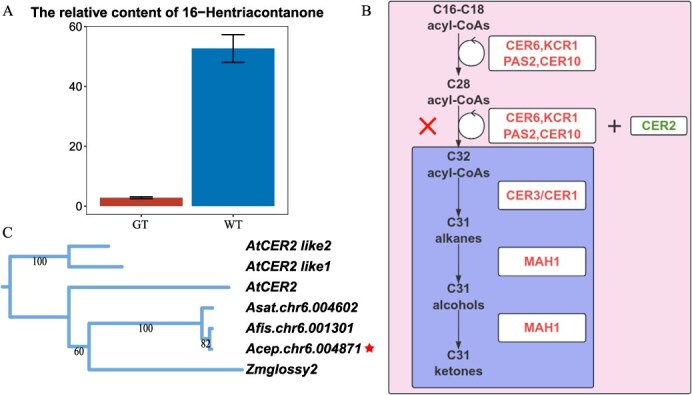
Insights into 16-hentriacontanone biosynthesis. (A) The bar plot of the relative content of 16-hentriacontanone. (B) Schematic overview of the 16-hentriacontanone synthesis pathway in onions: genes functioning correctly are indicated on the left side of the plus sign, mutated genes are highlighted on the right side of the plus sign, and the synthesis processes that are impaired are enclosed within a box below the cross symbol. (C) Phylogenetic analysis of *CER2* genes in the onion, maize, and *A. thaliana*

To test our hypothesis, we performed gas chromatography–mass spectrometry (GC–MS) analysis on the cuticles from leaves of both WT and GT onions. The results demonstrated a notable reduction in 16-hentriacontanone in the GT group (Fig. 3a), confirming that the glossy phenotype is caused by the absence of 16-hentriacontanone. Building upon existing research [29], we outlined the biosynthetic pathway for 16-hentriacontanone in onions and mapped all the necessary enzymes in the onion genome (Fig. 3b, Fig. S9). In onions, most of the key enzymes involved in the biosynthesis of VLCFAs were present in multiple copies, reflecting the importance of VLCFAs as precursors for various secondary metabolites and plant hormones essential for plant growth. An exception was found in the case of *CER10*, which encodes enoyl-CoA reductase and is present as a single copy. In contrast, the genes *CER1* and *CER3*, responsible for encoding aldehyde decarbonylase in the alkane synthesis process, do not show gene duplication in onions. Nevertheless, tandem duplication of these genes has been observed in other *Allium* species (*Allium fistulosum* and *Allium sativum* L.). Furthermore, *MAH1* gene, crucial for hydroxylation processes, also showed tandem duplication across these three *Allium* species (Fig. S9).

Interestingly, although several genes in the pathway showed polymorphisms, the frequencies of these variants did not conform to the characteristics of recessive inheritance except for the premature stop codon mutation in *AcCER2*. In addition, three copies of the *CER2* gene in *A. thaliana* all match the same single onion gene (*Acep.chr6.004871*), indicating no redundancy of *CER2* in the onion genome (Fig. 3c). Further phylogenetic analysis in several monocots confirmed that *CER2* genes were consistently found as a single copy in *Allium* plants including onion, Welsh onion, and garlic (Fig. S10). These findings led us to conclude that the primary cause of the absence of EW in onions is the mutation in *AcCER2*, which disrupts the elongation of VLCFAs from 28C to 32C. This disruption hinders the biosynthesis of 16-hentriacontanone, resulting in the observed glossy phenotype.

### Transcriptome comparison between GT and WT accessions identified coexpression module of *AcCER2* enriched in wax biosynthesis function

**Figure 4:**
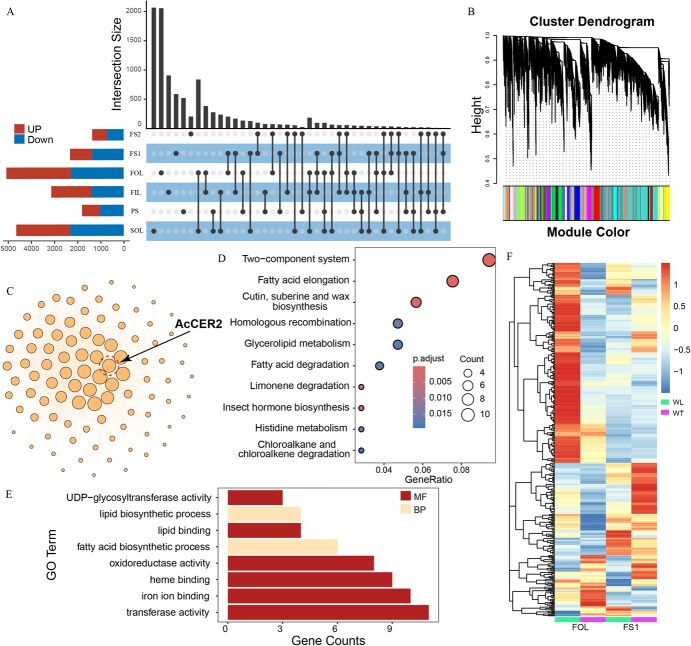
Transcriptome analysis of WT and GT onions. (A) An upset plot of DEGs between WT and GT accessions across different tissues and stages. The Upset plot illustrated the intersections and unique occurrences of DEGs in different tissue samples. Each column represents a unique combination of tissues, with the size of the dot indicating the number of DEGs shared between the tissue types. The horizontal bars on the left depicted the total number of DEGs in each tissue, while the vertical bars showed the count of DEGs within each intersection of tissues. (B) Cluster dendrogram from WGCNA Analysis. This dendrogram represents the hierarchical clustering of modules identified by the WGCNA package. Each branch of the dendrogram corresponds to a module of coexpressed genes. The color band below the dendrogram assigns a unique color to each module, facilitating their differentiation. The height of the merge (Y-axis) indicates the degree of similarity between modules, with lower merge heights representing greater similarity. (C) Network Interaction Diagram within the Gene Module Containing *AcCER2*. Each node represents a gene, with *AcCER2* centrally positioned, and the edges denote significant coexpression relationships (weight >0.3). (D) The bubble plot of significant KEGG pathways enriched in genes in the module containing *AcCER2*. Each bubble represents a distinct pathway, with its size indicating the gene counts and the color denoting the *P*-value, reflecting the statistical significance of the enrichment. (E) The bar plot of significant GO terms enriched in genes in the module containing *AcCER2*. (F) The heat map of gene expressions in the module containing *AcCER2*.

For the investigation of transcriptional regulation underlying phenotypic differences in EW accumulation in onions, RNA-seq was conducted across various tissues and developmental stages in both WT and GT accessions, including outer leaves of seedlings (SOL), pseudostem of seedlings (PS), inner leaves of flowering onions (FIL), outer leaves of flowering onions (FOL) (Fig. S11), the top portion of the flowering stalk (FS1), and the base portion of the flowering stalk (FS2). The largest number of differentially expressed genes (DEGs) was found in FOL, totaling 4975 DEGs, with 2238 downregulated and 2737 upregulated genes in GT compared to WT accessions (Fig. 4a). SOL also exhibited a substantial number of DEGs, totaling 4557, including 837 genes differentially expressed in both developmental stages (Fig. 4a). FS2 showed the fewest DEGs, likely due to the absence of distinct EW at the base portion of the flowering stalk in both WT and GT accessions (Fig. 4a). Notably, the gene expression level of *AcCER2* in GT accessions was significantly lower than that in WT accessions across all tissues and developmental stages used in our study, and we speculated that non-sense-mediated mRNA decay (NMD) could be a plausible explanation for this phenomenon (Fig. S3b).

The phenotypic differences between WT and GT accessions were most pronounced in FOL and FS1 (Fig. S12). Therefore, Gene Ontology (GO) and Kyoto Encyclopedia of Genes and Genomes (KEGG) pathway enrichment analyses were performed on DEGs identified in these tissues. In FOL, DEGs were enriched in 45 GO terms, including those related to signal transduction (e.g. protein kinase activity, protein phosphatase inhibitor activity) and transfer activities (e.g. transferring glycosyl groups, xyloglucosyl transferase activity) (Fig. S13). These changes likely reflected the adaptation of onions to various abiotic stresses due to the absence of EW. DEGs in FOL were also enriched in GO terms related to transmembrane transport and lipid metabolic processes, directly contributing to EW accumulation. Similar results were observed in FS1, with significant enrichment in lipid metabolic process, lipid transport, and cell wall modification terms (Fig. S14). Both FOL and FS1 showed enrichment in genes related to photosystem II, possibly due to increased light absorption efficiency in GT accessions with EW defect, which reshaped gene expression patterns in the photosynthesis system (Figs S13 and S14).

Cell wall structure serves as the primary defense against external abiotic and biotic stresses. Prior research has shown that plants can mitigate insect infestations by remodeling cell wall components [30]. In our study, DEGs in both FOL and FS1 were significantly enriched in xyloglucosyl transferase and pectinesterase, which were crucial for cell wall composition (Fig. S15). It could represent an alternative mechanism by which GT accessions develop resistance to thrips.

To assess the impact of *AcCER2* on gene expression regulation across onion tissues and developmental stages, a weighted gene coexpression network (WGCN) was constructed. After filtering out unexpressed and insignificantly fluctuating genes, 22 779 genes were categorized into 44 coexpression modules (Fig. 4b). The module containing *AcCER2* comprised 387 genes, and *AcCER2* showing direct coexpression relationships with 76 genes (Fig. 4c). These genes were differentially expressed between GT and WT accessions and exhibited tissue-specific expression patterns (Fig. 4f).

GO terms related to lipid synthesis and fatty acid synthesis processes were significantly enriched in genes of the *AcCER2* module (Fig. 4e). Similarly, also enriched in these genes, were KEGG pathways associated with fatty acid elongation, as well as cutin, suberine, and wax biosynthesis, with a concentration of genes associated with 3-ketoacyl-CoA synthase, key for elongating VLCFAs (Fig. 4d). This enzyme catalyzes the addition of two-carbon units to fatty acid chains, a process crucial for extending fatty acid chain length [7, 31]. Additionally, significant enrichment was observed for genes involved in the category of ATP-binding cassette, responsible for transmembrane lipid transport (Fig. 4d). Significantly, pectinesterase category were also enriched, echoing the findings in the DEGs enrichment analysis. Previous research has demonstrated that the suppression of pectin methylesterase (PME) activity can decrease aphid attraction and feeding behavior in plants, thereby bolstering insect resistance [32]. This aligns with a plausible mechanism that might contribute to the thrip resistance observed in GT accessions.

### Metabolomic analysis showed the increase in flavonoids and the reduction in soluble sugars may contribute to insect resistance

**Figure 5:**
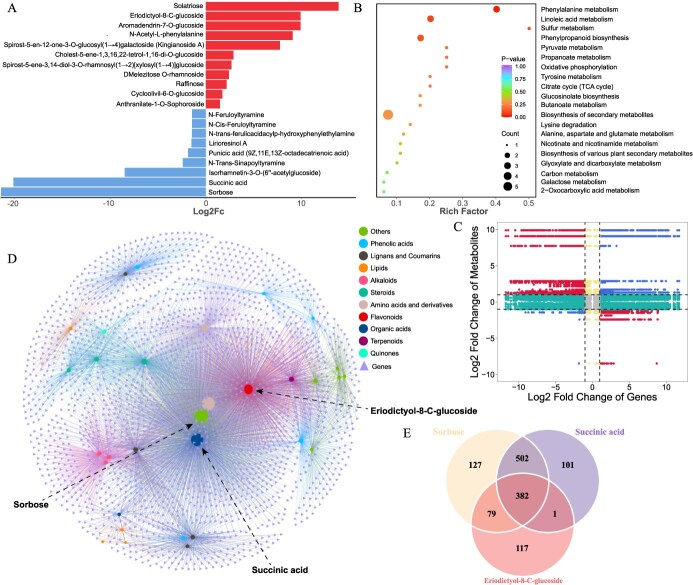
Comprehensive metabolomic and gene expression analysis in WT and GT onions. (A) Top 20 Metabolites exhibiting significant difference in content between GT and WT accessions. The length of each column represents the log2 fold change of metabolite levels in GT over that in WT. Metabolites on the right side of the central axis represent metabolites with significant upregulation, while those on the left side represent metabolites with significant downregulation. (B) The bubble plot of significant KEGG pathways enriched in DAMs. Each bubble represents a distinct pathway, with the size indicating the count of metabolites and the color denoting the *P*-value, reflecting the statistical significance of the enrichment. (C) Nine quadrant analysis of DAMs and DEGs identified between WT and GT accessions. The plot was divided into nine quadrants by dashed lines, with the quadrants numbered from left to right, top to bottom (1 to 9). (D) The network based on the correlation between gene expression levels and metabolite accumulation, where each triangle represents a gene, each circle represents a metabolite, and different colors reflect different classes of metabolites. (E) The Venn diagrams illustrated the overlap and unique associations of genes that exhibit significant correlations with each of the three specified metabolites.

In an effort to identify specific metabolic alterations that confer thrip resistance in GT accessions, comprehensive metabolomic analyses were conducted on both GT and WT accessions. Principal component analysis (PCA) and clustering based on metabolite profiles revealed distinct metabolic differences between the WT and GT groups (Fig. S16). Analysis of metabolites of flowering stalk indicated 37 differentially abundant metabolites (DAMs), with 14 significantly downregulated and 23 upregulated in GT accessions (Table S4). Notably, Sorbose and Succinic acid were the most reduced, whereas Solatriose and Eriodictyol-8-C-glucoside exhibited the greatest increase in the GT group (Fig. 5a). Orthogonal Partial Least Squares Discriminant Analysis (OPLS-DA) also highlighted high variable importance in projection (VIP) values for Eriodictyol-8-C-glucoside, Sorbose, and Succinic acid among the top five, though Solatriose did not feature in these high-VIP metabolites (Fig. S17). An enrichment analysis of these DAMs in KEGG pathways showed significant overrepresentation in the biosynthesis of secondary metabolites pathway (Fig. 5b), a crucial defense against various biotic stresses, suggesting that GT accessions may attain thrip resistance through the regulation of secondary metabolite biosynthesis.

Furthermore, we analyzed the correlation between metabolite content and gene expression (r > 0.9, *P* < .01), visualizing the results in a nine-quadrant plot showing differential expression and abundance. Due to complex post-transcriptional modifications in biological systems, most points were clustered in quadrants 2, 4, 5, 6, and 8, indicating a lower correlation between gene expression levels and metabolite accumulation. Points in quadrants 1 and 9 showed a significant positive correlation between DAMs and DEGs, whereas those in quadrants 3 and 7 indicated a negative correlation (Fig. 5c). These points were further utilized to construct a correlation network between DEGs and DAMs. Employing the Fruchterman–Reingold algorithm for network visualization revealed that Eriodictyol-8-C-glucoside, Sorbose, and Succinic acid held central positions in the network, correlating with numerous genes (Fig. 5d). These findings imply that these metabolites might be key contributors to the observed differences in onion resistance to thrips.

Accordingly, we proposed the hypothesis that the primary feeding preference of thrips for the nutrient-rich sap from onion leaves and stalks may be modulated by these metabolic alterations. The reduction in Sorbose, a type of soluble sugar, could potentially make onions less appealing to thrips. Additionally, flavonoid compounds, such as Eriodictyol-8-C-glucoside, may serve as repellents to thrips, thereby functioning as a deterrent. Moreover, statistical analysis of genes correlated with these three key metabolites identified 382 genes associated with all of them (Fig. 5e). Intriguingly, *AcCER2* was included among these genes, suggesting its potential direct influence on the metabolism of flavonoids and soluble sugars in onions, thereby contributing to thrip resistance.

## Discussion

Unlike the majority of plants, the defect in EW in glossy mutants of onions contributes to an increased resistance of their leaves and stems against thrips. Thus, it is critically important to explore the regulatory mechanisms behind the glossy phenotype in onions and to elucidate the fundamental reasons why the defect of EW confers enhanced thrip resistance in these plants. Consistent with previous research [25, 26], the segregation ratio of GT and WT progeny in this study confirmed that the trait is governed by a single recessive gene. Benefiting from the construction of a high-quality reference genome [33], we were able to identify a multitude of variant sites in a high-throughput manner. This advancement coupled with bulked segregation analysis enabled us to precisely pinpoint the gene regulating the trait.

Prior study revealed a candidate region on chromosome 5 [24], and we discerned this segment spanning 1261.67 to 1419.83 Mb on chromosome 5 by aligning the candidate interval pinpointed through earlier genetic mapping efforts onto the reference genome of the onion. Within this range, *Acep.chr5.004607*, *Acep.chr5.004623*, *Acep.chr5.004624*, and *Acep.chr5.004894* were identified as MYB TFs (transcription factors), and *Acep.chr5.005008* as an HD-ZIP TF. Homologs of these genes in *A. thaliana* (*AT4G28110* [34], *AT3G47600* [35], *AT3G28910* [36], *AT5G15310* [37], *AT3G61150* [38]) have been documented to play roles in the regulation of fatty acid or epidermal wax biosynthesis. Recently, a single locus named *gl^ogl^* at position 113.1 cM on chromosome 6 related to the glossy phenotype was reported [39]. In order to validate our results, we mapped the flanking genetic markers i32152_614 and i32739_152 (110.9 and 132.4 cM on chromosome 6) to the reference genome (1107.66 and 1660.00 Mb on chromosome 6) and estimated the genomic location of the *gl^ogl^* to be ~1147 Mb on chromosome 6, based on the genetic and physical distances between these markers. This region not only fell within our initially identified candidate interval but was also very close to *AcCER2* (start position: 1155654637). Considering the margin of error in distance estimation, our results are highly consistent with previous study, which strongly support the reliability of our findings. To summarize, we hypothesized that the EW of onions is a pivotal element in the plant’s adaptation to environmental changes. This adaptation necessitates a sophisticated gene regulatory network tasked with regulating the biosynthesis of EW. Variations in the genes within these regulatory modules are correlated with corresponding changes in EW content, which are generally gradual. Conversely, mutations in genes responsible for encoding crucial enzymes needed for EW biosynthesis may result in a significant reduction, and are exhibited as qualitative hereditary traits.

The *CER2* was involved in the elongation of VLCFAs from C28 to C32, and has been shown to be essential for biosynthesis of EW in *A. thaliana* [40], apple [41], rice [42], and Chinese cabbages [43]. In *A. thaliana*, plants with *AtCER2* mutations display a glossy phenotype and notably lack VLCFAs with length of carbon chains extending beyond 28 [8]. In this study, we discovered that an AA insertion is present in *AcCER2* in all of GT accessions, leading to a frameshift mutation in the amino acid sequence encoded by this gene, resulting in premature termination. GC–MS analysis revealed a significant reduction in the content of 16-hentriacontanone in GT accessions. We speculated that this is due to the mutation in *AcCER2*, leading to a deficiency of 32C fatty acyl-CoA substrates in GT accessions.

A diverse array of plant secondary metabolites plays a crucial role in adaptation and defense against herbivorous threats [44, 45]. These metabolites can act as repellents, deterrents, growth inhibitors, or cause direct mortality to herbivores [46]. Numerous previous studies have consistently demonstrated that the enhanced accumulation of various flavonoids in plants significantly strengthens their resistance against insects [47–49]. This increased resistance was attributed to the ability of flavonoids to act as natural deterrents or toxins, effectively reducing insect feeding and infestation [49]. Additionally, flavonoids play a pivotal role in the plant’s defensive signaling pathways, further enhancing their ability to ward off insect attacks [47]. Notably, the accumulation of Eriodictyol-8-C-glucoside, a flavonoid, was significantly increased in GT accessions, suggesting that this metabolite may be able to improve plant insect resistance as well as previously reported flavonoids. Furthermore, plants can enhance their defense against insects by adjusting the structure of cell walls, and this modification process is often activated by plant hormone signaling pathways [50–52]. Therefore, the observed changes in the pectinesterase and xyloglucan xyloglucosyl transferases between WT and GT accessions might reflect a rebuilding of the cell wall. Meanwhile, the decrease in soluble sugar accumulation in GT accessions might also affect the allure of onion plants to thrips. Consequently, the property of thrip resistance in GT accessions might result from multiple physiological alterations. The specific changes responsible for this feature remain to be identified through further research.

Although the function of *CER2* in plants has been extensively and thoroughly investigated [53, 54], to date, there have been no reports linking this gene with insect resistance. Are the changes in the series of characteristics in GT accessions due to the absence of EW, which alters the effects of external environmental factors such as temperature, humidity, and light on the plant, thereby impacting its physiological traits? Or, are these changes akin to the biosynthesis of plant EW also influenced by the *AcCER2* mutation, resulting in alterations in the gene regulatory network related to thrip resistance? The construction of gene expression network seemed to provide some evidence that *AcCER2* itself is involved in the biological processes related to onion thrip resistance, as the gene expression module it belongs to was both involved in lipid synthesis and transport, cell wall modification, and signal transduction. Moreover, the integration of transcriptomic and metabolomic data also indicated a significant correlation between the gene expression level of *AcCER2* and the accumulation of flavonoids and soluble sugars, which further support our conjecture.

It is noteworthy that while GT accessions exhibit resistance to thrips, the absence of EW may lead to reduced tolerance of onion plants to abiotic stress [2, 3, 55, 56]. Therefore, a deeper exploration of the regulatory mechanisms behind the lack of EW and thrip resistance is vital for breeding of onions with both excellent agronomic performance and resistance to thrips, which will be the primary focus of our future work. In summary, this study utilized whole-genome resequencing for rapid genotyping of WT and GT accessions and accurately identified the key gene *AcCER2* involved in the biosynthesis of onion EW, providing new theoretical information for researches related to onion wax biosynthesis. By integrating transcriptomic and metabolomic data, we preliminarily explained the relationship between the onion GT phenotype and property of thrip resistance, which has significant implications for onion breeding.

## Materials and methods

### Plant materials and trait measurement

All onion materials used in this study were intermediate-day types and were sourced from the Vegetable Research Department of the Lianyungang Academy of Agricultural Sciences. In November 2017, GT accessions were selected from the resource nursery. Then, Onion bulbs were harvested in May 2018 and planted in October. In May 2019, individual plants were bagged for self-pollination during the flowering period. After that, the harvested seeds were sown in September and transplanted in November. Twenty GT accessions were managed with normal fertilization and watering, with either standard pesticide applications or no pesticide use throughout the growth period. As controls, three yellow-skinned and three purple-skinned waxy onion accessions were selected and managed with same treatments. In April 2020, plant height at the onset of bulb swelling and thrip counts were recorded, followed by maturity period and average single-bulb weight in late May. During the final phase of bulb swelling, the damage to onion leaves by thrips was observed. For the 19 061 self-pollinated WT and GT mutant accessions, manual emasculation and assisted pollination were employed for crossbreeding. The harvested F1 generation was self-pollinated, and the segregation ratio in the F2 generation was analyzed. Samples used for validation, RNA sequencing, and metabolomic analysis were grown with standard fertilization and watering, without any insecticides applied throughout the growth period.

### Ultrastructural observation of onion leaf surfaces

Leaf samples from 19 061 accessions, both with and without EW, were prepared for ultrastructural observation. The leaves were transversely cut into sections measuring 5 × 5 mm. These sections were then fumigated with 2% osmium tetroxide for 24 h and air-dried naturally for another 24 h. The samples were fixed onto the specimen stage using silver adhesive, followed by gold sputtering. Observations were then conducted under a Scanning Electron Microscope (SEM).

### Evaluations of EW composition

Leaves from WT and GT accessions were submerged in 15 ml of hexane in vials for 30 s to extract waxes, respectively. Then, 20 μg of C24 alkane (SUPELCO, Sigma) was added to each sample as an internal standard, followed by drying under nitrogen gas. Next, 100 μl of N,O-Bis(trimethylsilyl) trifluoroacetamide (BSTFA, SUPELCO, Sigma) was added, and the samples were derivatized in a 90°C incubator for 30 min. After drying the derivatizing agent with nitrogen gas, 1 ml of hexane was added to dissolve the samples, which were then filtered and transferred to new chromatography vials. The samples were analyzed using a Gas Chromatography–Mass Spectrometer (Thermo Trace1310 ISQ). Automatic retrieval of mass spectral data for each component was conducted, and the results were verified against standard spectra and relevant literature to identify the components. The content of each component was calculated using the area normalization method.

### Whole-genome sequencing of bulked DNA

The F2 individuals were categorized into WT and GT groups based on their EW. From each group, 30 plants were randomly selected to construct mixed sample pools. For each DNA pool, genomic DNA was extracted from the fresh leaf tissue of the selected F2 individuals. This extraction utilized the DNAprep Pure Plant Kit (Magen). Approximately 0.05 g of fresh leaves from each individual in the pool were combined in a mortar for homogenization. The genomic DNA was then extracted from this homogenized leaf sample for further sequencing analysis.

The extracted genomic DNA underwent ultrasonic fragmentation to achieve an optimal size range for sequencing. A sequencing library was subsequently constructed with a targeted insert size of 400–500 bp using the MGIEasy DNA Library Prep Kit. Post-library construction, the DNA samples underwent purification using a silica membrane column. Size selection of the library was carried out through agarose gel electrophoresis to ensure the correct fragment size distribution. The final DNA library for each pool was sequenced using the BGI T7 sequencing system.

### Variants calling and annotation for each pool

After conducting quality control and filtering of the raw sequencing data with fastp software [57], the resulting clean reads were aligned to the reference genome using BWA software [58]. Variant detection was then performed in accordance with the Best Practices workflow as officially recommended by GATK [59]. Following the removal of low-confidence variants and those appearing as homozygous alternate in both pools, each variant site was functionally annotated using SnpEff [60].

### Identification of candidate region associated with glossy phenotype

Initially, we filtered out variant sites with a depth (DP) <25 or >250. Then, we calculated allelic frequencies using ED, followed by sliding window analysis (with a window size of 1 Mb and a step size of 100 kb) [61], to identify regions where target variants are located. To validate our identification results, we also employed other classic identification methods, including the standard G-statistic [62], allele frequency method [63], and significant structural variant method [64].

After preliminarily identifying candidate regions and considering that our target trait is a qualitative trait controlled by a single gene, we further filtered out variants that appeared heterozygous in both pools. We also eliminated variants in intergenic regions to reduce the impact of repetitive sequences on our identification results. Subsequently, we characterized the likelihood of a variant site being a candidate based on the ED between the allelic frequencies of each variants and the ‘ideal frequencies’ (homozygous in the GT pool and one-third consistent, two-thirds inconsistent with the GT pool in the WT pool). We then selected the variants nearest to the ‘ideal frequencies’ (top 5%) as our final candidate sites.

### Sequence validation of *AcCER2* in other onion accessions

RNA from the validation samples was isolated using the Plant RNA Kit (Omega Bio-Tek, USA) according to the manufacturer’s instructions and subsequently reverse-transcribed into cDNA. The *AcCER2* gene was then amplified from the cDNA of each validation samples, and the resulting *AcCER2* sequences were obtained through Sanger sequencing. Finally, multiple sequence alignment was performed using MAFFT software [65] to identify mutation sites.

### Identification of genes encoding enzymes essential for 16-hentriacontanone biosynthesis

To identify genes in onions encoding key enzymes of the FAE complexes and decarbonylation (alkane-forming) pathway, we used already identified genes as queries in *A. thaliana*, rice, and maize, including *AtKCS1, AtKCS6, AtKCR1, AtCER10, AtPAS1, AtMAH1, AtCER1, AtPAS2, AtCER3, OsWSL1, OsWSL3, Zmglossy2, Zmglossy4, Zmglossy8, AtCER2-like1, AtCER2-like2,* and *AtCER2*. We conducted a blast [66] analysis against all onion protein sequences to find the best hits. Furthermore, the presence of conserved domains in these enzymes was confirmed through HMMER web server [67].

### Identification of DEGs

We performed transcriptome sequencing to analyze gene expression across various tissues and developmental stages in both WT and GT accessions (grown under normal fertilization and watering conditions, with no pesticide use throughout the growth period.). The tissues selected for this study included SOL, PS, FIL, FOL, FS1, and FS2. For each tissue type, three biological replicates were prepared to ensure robustness in our data.

Total RNA was extracted from each tissue sample using the RNAprep Pure Plant Kit (Tiangen Biotech), ensuring high-quality RNA suitable for next-generation sequencing. Library preparation for sequencing was conducted using the MGIEasy RNA Library Prep Kit (MGI), following the manufacturer’s instructions. Finally, sequencing was carried out on the BGI T7 sequencing platform.

After filtering the raw reads using fastp [57], they were aligned to the reference genome using Hisat2 [68]. The resulting SAM files were then sorted and converted into BAM files using Samtools [69]. Subsequently, gene expression levels were quantified using FeatureCounts [70]. Differential expression analysis was performed using DESeq2 [71], and genes with a fold change >2 or less than −2, along with an adjusted *P*-value < 0.05, were identified as DEGs. To explore the functional implications of these DEGs, we utilized the clusterProfiler package in R software [72], conducting GO and KEGG pathway enrichment analyses.

### Construction of coexpression network

We utilized weighted gene coexpression network analysis (WGCNA) [73] on prefiltered expression data from all tissues and developmental stages of onions used in this study to identify gene modules with distinct expression patterns. The soft-thresholding power was set at 8, adhering to the criterion of approximate scale-free topology, to construct an unsigned network. The minimum module size was set to 30, ensuring the biological relevance of the modules. Module merging was guided by a dissimilarity threshold set at 0.25, allowing for the combination of similar expression profiles while maintaining distinct modules for significantly different patterns.

### Extensive metabolomics analysis

We conducted metabolomic analysis of the top portion of the flowering stalk in both WT and GT accessions using ultra-high-performance liquid chromatography–tandem mass spectrometry (UPLC-MS/MS). The experiment included three biological replicates for both WT and GT accessions, with all samples grown under normal fertilization and watering conditions, and no pesticide use throughout the growth period. Data acquisition and validation were efficiently handled using Analyst software (version 1.6.3; AB Sciex).

### Identification of DAMs

OPLS-DA was performed using the OPLSR. Anal function in the MetaboAnalystR package within R software [74]. Metabolites in WT and GT accessions with a fold change >2 or less than −2, and a VIP value >1, were identified as DAMs. Subsequently, these DAMs were used for KEGG pathway enrichment analysis using the MetaboAnalystR package [74].

### Statistical analysis

All statistical analyses were conducted in R 4.3.2.

## Supplementary Material

Web_Material_uhaf006

## Data Availability

All raw sequencing data generated in this study have been deposited to CNSA (https://db.cngb.org/cnsa/) under accession CNP0005428.
