# Supplementary material for: A premature termination codon mutation in the onion AcCER2 gene is associated with both glossy leaves and thrip resistance
Source: Hortic Res. 2025 Jan 14;12(4):uhaf006. doi: 10.1093/hr/uhaf006 (PMC11896967; doi:10.1093/hr/uhaf006)
Supplement: Web_Material_uhaf006 [file web_material_uhaf006.zip › Figure S4.pdf]

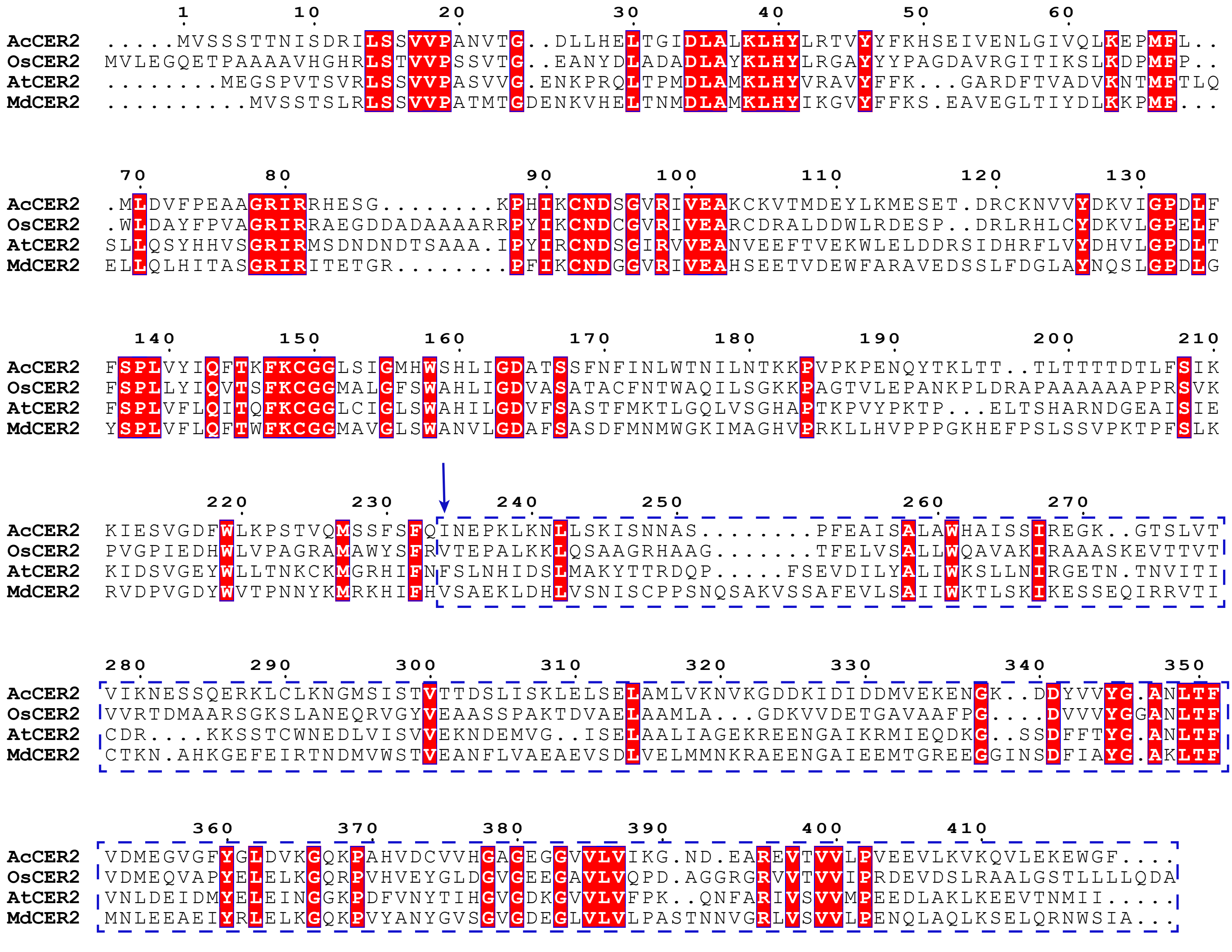
1 10 20 30 40 50 60
AcCER2 .....MVSSSTTNISDRILSSVVPANVTG..DLLHELTGIDLALKLHYLRTVYYFKHSEIVENLGIVQLKEPMFL..
OsCER2 MVLEGQETPAAAAVHGHRLSTVVPSSVTG..EANYDLADADLAYKLHYLRGAYYYPAGDAVRGITIKSLKDPMFP..
AtCER2 ........MEGSPVTSVRLSSVVPASVVG.ENKPRQLTPMDLAMKLHYVRAVYFFK...GARDFTVADVKNTMFTLQ
MdCER2 ..........MVSSTSLRLSSVVPATMTGDENKVHELTNMDLAMKLHYIKGVYFFKS.EAVEGLTIYDLKKPMF...
70 80 90 100 110 120 130
AcCER2 .MLDVFPEAAGRIRRHESG........KPHIKCNDSGVRIVEAKCKVTMDEYLKMESET.DRCKNVVYDKVIGPDLF
OsCER2 .WLDAYFPVAGRIRRAEGDDADAAARRPYIKCNDCGVRIVEARCDRALDDWLRDESP..DRLRHLCYDKVLGPELF
AtCER2 SLLQSYHHVSGRIRMSDNDNDTSAAA.IPYIRCNDSGIRVVEANVEEFTVEKWLELDDRSIDHRFLVYDHVLGPDLT
MdCER2 ELLQLHITASGRIRITETGR........PFIKCNDGGVRIVEAHSEETVDEWFARAVEDSSLFDGLAYNQSLGPDLG
140 150 160 170 180 190 200 210
AcCER2 FSPLVYIQFTKFKCGGLSIGMHWSHLIGDATSSFNFINLWTNILNTKKPVPKPENQYTKLTT..TLTTTTDTLFSIK
OsCER2 FSPLLYIQVTSFKCGGMALGFSWAHLIGDVASATACFNTWAQILSGKKPAGTVLEPANKPLDRAPAAAAAPPRSVK
AtCER2 FSPLVFLQITQFKCGGLCIGLSWAHILGDVFSASTFMKTLGQLVSGHAPTKPVYPKTP...ELTSHARNDGEAISIE
MdCER2 YSPLVFLQFTWFKCGGMAVGLSWANVLGDAFSASDFMNMWGKIMAGHVPRKLLHVPPPGKHEFPSLSSVPKTPFSLK
220 230 240 250 260 270
AcCER2 KIESVGDFWLKPSTVQMSSFSFQINEPKLKNLLSKISNNAS........PFEAISALAWHAISSIREGK..GTSLVT
OsCER2 PVGPIEDHWLVPAGRAMAWYSFRVTEPALKKLQSAAGRHAAG.......TFELVSALLWQAVAKIRAAASKEVTTVT
AtCER2 KIDSVGEYWLLTNKCKMGRHIFNFSLNHIDSLMAKYTTRDQP.....FSEVDILYALIWKSLLNIRGETN.TNVITI
MdCER2 RVDPVGDYWVTPNNYKMRKHIFHVSAEKLDHLVSNISCPPSNQSAKVSSAFEVLSAIIWKTLSKIKESSEQIRRVTI
280 290 300 310 320 330 340 350
AcCER2 VIKNESSQERKLCLKNGMSISTVTTDSLISKLELSELAMLVKNVKGDDKIDIDDMVEKENGK..DDYVVYG.ANLTF
OsCER2 VVRTDMAARSGKSLANEQRVGYVEAASSPAKTDVAELAAMLA...GDKVVDETGAVAAFPG....DVVVYGGANLTF
AtCER2 CDR....KKSSTCWNEDLVISVVEKNDEMVG..ISELAALIAGEKREENGAIKRMIEQDKG..SSDFFTYG.ANLTF
MdCER2 CTKN.AHKGEFEIRTNDMVWSTVEANFLVAEAEVSDLVELMMNKRAEENGAIEEMTGREEGGINSDFIAYG.AKLTF
360 370 380 390 400 410
AcCER2 VDMEGVGFYGLDVKGQKPAHVDCVVHGAGEGGVLVIKG.ND.EAREVTVVLPVEEVLKVKQVLEKEWGF....
OsCER2 VDMEQVAPYELELKGQRPVHVEYGLDGVGEEGAVLVQPD.AGGRGRVVTVVIPRDEVDSLRAALGSTLLLLQDA
AtCER2 VNLDEIDMYELEINGGKPDFVNYTIHGVGDKGVVLVFPK..QNFARIVSVVMPEEDLAKLKEEVTNMII.....
MdCER2 MNLEEAEIYRLELKGQKPVYANYGVSGVGDEGLVLVLPASTNNVGRLVSVVLPENQLAQLKSELQRNWSIA...
