# Supplementary material for: A premature termination codon mutation in the onion AcCER2 gene is associated with both glossy leaves and thrip resistance
Source: Hortic Res. 2025 Jan 14;12(4):uhaf006. doi: 10.1093/hr/uhaf006 (PMC11896967; doi:10.1093/hr/uhaf006)
Supplement: Web_Material_uhaf006 [file web_material_uhaf006.zip › Figure S6.pdf]

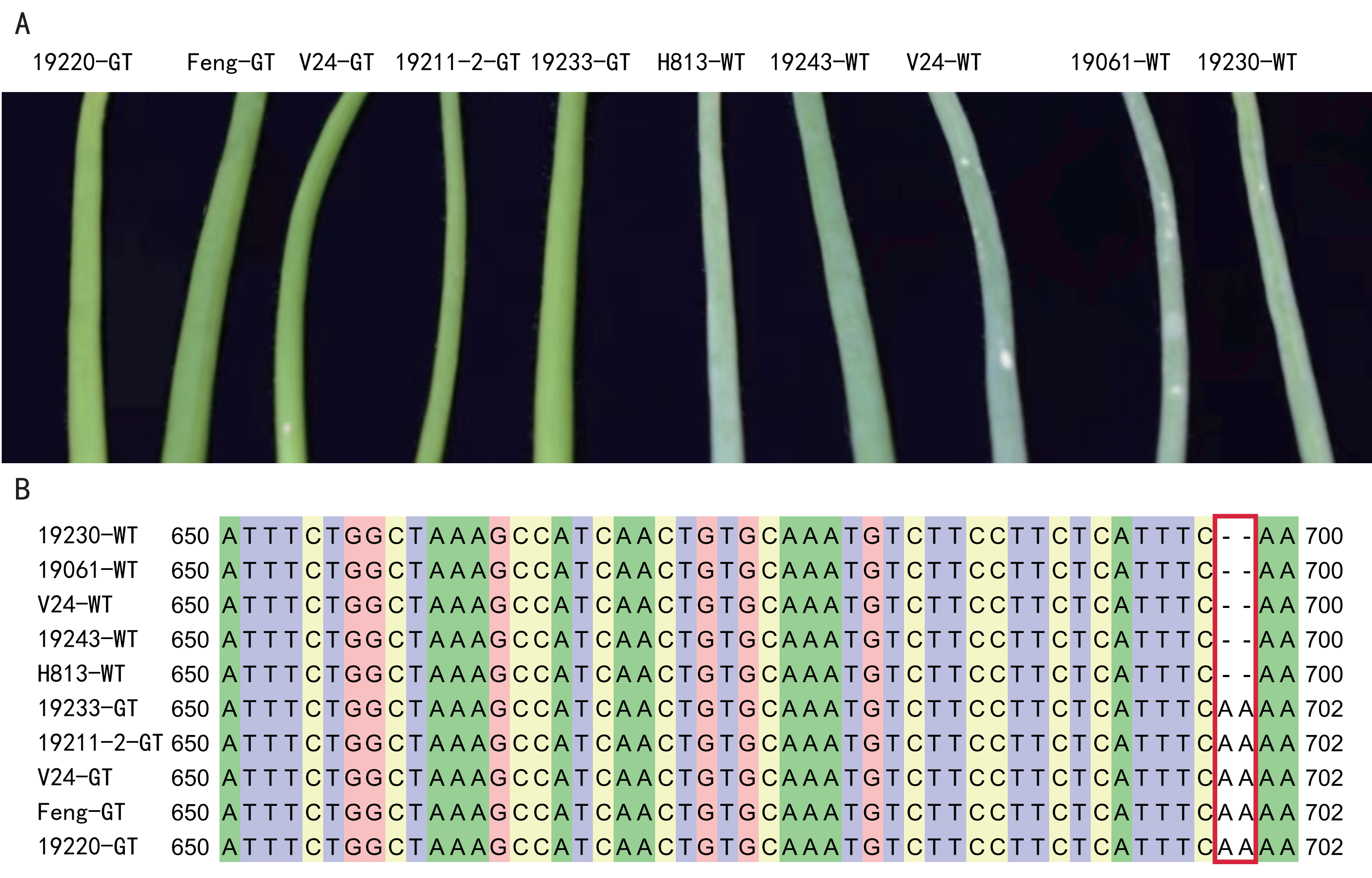
A
19220-GT
Feng-GT
V24-GT
19211-2-GT
19233-GT
H813-WT
19243-WT
V24-WT
19061-WT
19230-WT
B
19230-WT 650 ATTTCTGGCTAAAGCCATCAACTGTGCAAATGTCTTCCTTCTCATTTC--AA 700
19061-WT 650 ATTTCTGGCTAAAGCCATCAACTGTGCAAATGTCTTCCTTCTCATTTC--AA 700
V24-WT 650 ATTTCTGGCTAAAGCCATCAACTGTGCAAATGTCTTCCTTCTCATTTC--AA 700
19243-WT 650 ATTTCTGGCTAAAGCCATCAACTGTGCAAATGTCTTCCTTCTCATTTC--AA 700
H813-WT 650 ATTTCTGGCTAAAGCCATCAACTGTGCAAATGTCTTCCTTCTCATTTC--AA 700
19233-GT 650 ATTTCTGGCTAAAGCCATCAACTGTGCAAATGTCTTCCTTCTCATTTCAAAA 702
19211-2-GT 650 ATTTCTGGCTAAAGCCATCAACTGTGCAAATGTCTTCCTTCTCATTTCAAAA 702
V24-GT 650 ATTTCTGGCTAAAGCCATCAACTGTGCAAATGTCTTCCTTCTCATTTCAAAA 702
Feng-GT 650 ATTTCTGGCTAAAGCCATCAACTGTGCAAATGTCTTCCTTCTCATTTCAAAA 702
19220-GT 650 ATTTCTGGCTAAAGCCATCAACTGTGCAAATGTCTTCCTTCTCATTTCAAAA 702
