# Supplementary material for: A premature termination codon mutation in the onion AcCER2 gene is associated with both glossy leaves and thrip resistance
Source: Hortic Res. 2025 Jan 14;12(4):uhaf006. doi: 10.1093/hr/uhaf006 (PMC11896967; doi:10.1093/hr/uhaf006)
Supplement: Web_Material_uhaf006 [file web_material_uhaf006.zip › Figure S7.pdf]

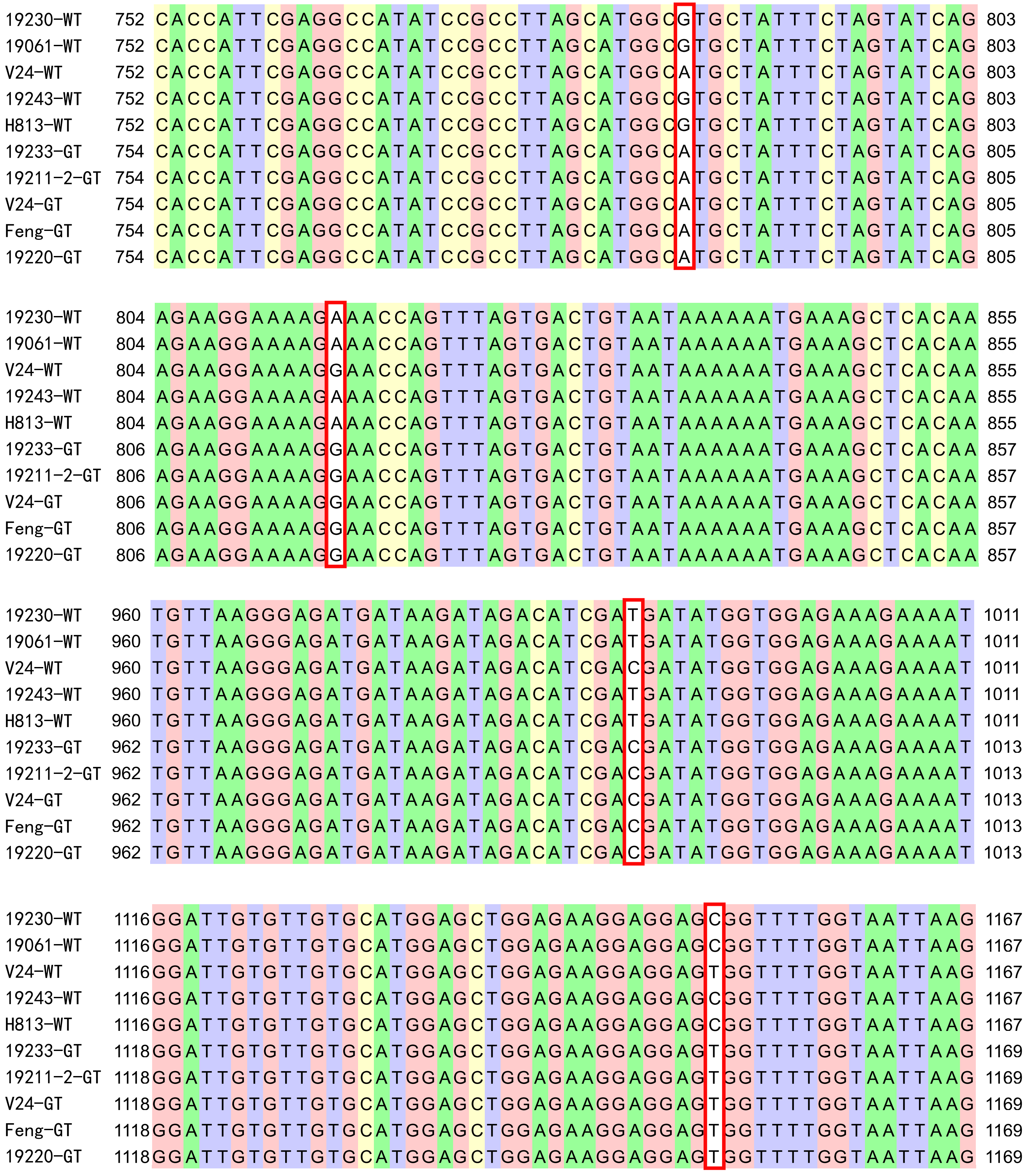
19230-WT 752 CACCATTCGAGGCCATATCCGCCTTAGCATGGCGTGCTATTTCTAGTATCAG 803
19061-WT 752 CACCATTCGAGGCCATATCCGCCTTAGCATGGCGTGCTATTTCTAGTATCAG 803
V24-WT 752 CACCATTCGAGGCCATATCCGCCTTAGCATGGCATGCTATTTCTAGTATCAG 803
19243-WT 752 CACCATTCGAGGCCATATCCGCCTTAGCATGGCGTGCTATTTCTAGTATCAG 803
H813-WT 752 CACCATTCGAGGCCATATCCGCCTTAGCATGGCGTGCTATTTCTAGTATCAG 803
19233-GT 754 CACCATTCGAGGCCATATCCGCCTTAGCATGGCATGCTATTTCTAGTATCAG 805
19211-2-GT 754 CACCATTCGAGGCCATATCCGCCTTAGCATGGCATGCTATTTCTAGTATCAG 805
V24-GT 754 CACCATTCGAGGCCATATCCGCCTTAGCATGGCATGCTATTTCTAGTATCAG 805
Feng-GT 754 CACCATTCGAGGCCATATCCGCCTTAGCATGGCATGCTATTTCTAGTATCAG 805
19220-GT 754 CACCATTCGAGGCCATATCCGCCTTAGCATGGCATGCTATTTCTAGTATCAG 805
19230-WT 804 AGAAGGAAAAGAAACCAGTTTAGTGACTGTAATAAAAAATGAAAGCTCACAA 855
19061-WT 804 AGAAGGAAAAGAAACCAGTTTAGTGACTGTAATAAAAAATGAAAGCTCACAA 855
V24-WT 804 AGAAGGAAAAGGAACCAGTTTAGTGACTGTAATAAAAAATGAAAGCTCACAA 855
19243-WT 804 AGAAGGAAAAGAAACCAGTTTAGTGACTGTAATAAAAAATGAAAGCTCACAA 855
H813-WT 804 AGAAGGAAAAGAAACCAGTTTAGTGACTGTAATAAAAAATGAAAGCTCACAA 855
19233-GT 806 AGAAGGAAAAGGAACCAGTTTAGTGACTGTAATAAAAAATGAAAGCTCACAA 857
19211-2-GT 806 AGAAGGAAAAGGAACCAGTTTAGTGACTGTAATAAAAAATGAAAGCTCACAA 857
V24-GT 806 AGAAGGAAAAGGAACCAGTTTAGTGACTGTAATAAAAAATGAAAGCTCACAA 857
Feng-GT 806 AGAAGGAAAAGGAACCAGTTTAGTGACTGTAATAAAAAATGAAAGCTCACAA 857
19220-GT 806 AGAAGGAAAAGGAACCAGTTTAGTGACTGTAATAAAAAATGAAAGCTCACAA 857
19230-WT 960 TGTTAAGGGAGATGATAAGATAGACATCGATGATATGGTGGAGAAAGAAAAT 1011
19061-WT 960 TGTTAAGGGAGATGATAAGATAGACATCGATGATATGGTGGAGAAAGAAAAT 1011
V24-WT 960 TGTTAAGGGAGATGATAAGATAGACATCGACGATATGGTGGAGAAAGAAAAT 1011
19243-WT 960 TGTTAAGGGAGATGATAAGATAGACATCGATGATATGGTGGAGAAAGAAAAT 1011
H813-WT 960 TGTTAAGGGAGATGATAAGATAGACATCGATGATATGGTGGAGAAAGAAAAT 1011
19233-GT 962 TGTTAAGGGAGATGATAAGATAGACATCGACGATATGGTGGAGAAAGAAAAT 1013
19211-2-GT 962 TGTTAAGGGAGATGATAAGATAGACATCGACGATATGGTGGAGAAAGAAAAT 1013
V24-GT 962 TGTTAAGGGAGATGATAAGATAGACATCGACGATATGGTGGAGAAAGAAAAT 1013
Feng-GT 962 TGTTAAGGGAGATGATAAGATAGACATCGACGATATGGTGGAGAAAGAAAAT 1013
19220-GT 962 TGTTAAGGGAGATGATAAGATAGACATCGACGATATGGTGGAGAAAGAAAAT 1013
19230-WT 1116 GGATTGTGTTGTGCATGGAGCTGGAGAAGGAGGAGCGGTTTTGGTAATTAAG 1167
19061-WT 1116 GGATTGTGTTGTGCATGGAGCTGGAGAAGGAGGAGCGGTTTTGGTAATTAAG 1167
V24-WT 1116 GGATTGTGTTGTGCATGGAGCTGGAGAAGGAGGAGTGGTTTTGGTAATTAAG 1167
19243-WT 1116 GGATTGTGTTGTGCATGGAGCTGGAGAAGGAGGAGCGGTTTTGGTAATTAAG 1167
H813-WT 1116 GGATTGTGTTGTGCATGGAGCTGGAGAAGGAGGAGCGGTTTTGGTAATTAAG 1167
19233-GT 1118 GGATTGTGTTGTGCATGGAGCTGGAGAAGGAGGAGTGGTTTTGGTAATTAAG 1169
19211-2-GT 1118 GGATTGTGTTGTGCATGGAGCTGGAGAAGGAGGAGTGGTTTTGGTAATTAAG 1169
V24-GT 1118 GGATTGTGTTGTGCATGGAGCTGGAGAAGGAGGAGTGGTTTTGGTAATTAAG 1169
Feng-GT 1118 GGATTGTGTTGTGCATGGAGCTGGAGAAGGAGGAGTGGTTTTGGTAATTAAG 1169
19220-GT 1118 GGATTGTGTTGTGCATGGAGCTGGAGAAGGAGGAGTGGTTTTGGTAATTAAG 1169
