# Supplementary material for: A premature termination codon mutation in the onion AcCER2 gene is associated with both glossy leaves and thrip resistance
Source: Hortic Res. 2025 Jan 14;12(4):uhaf006. doi: 10.1093/hr/uhaf006 (PMC11896967; doi:10.1093/hr/uhaf006)
Supplement: Web_Material_uhaf006 [file web_material_uhaf006.zip › Supplementary data.docx]

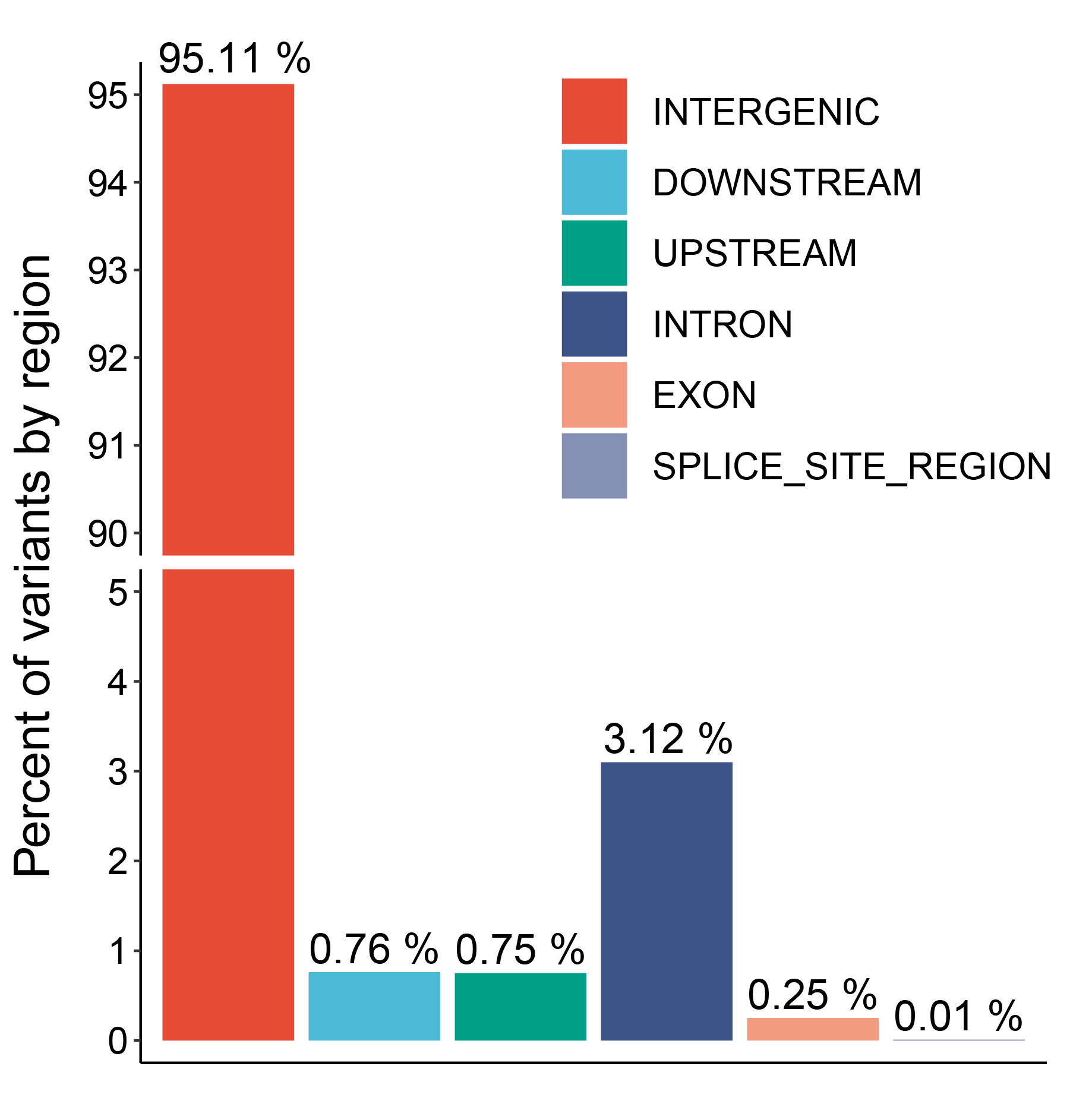


**Figure S1** The bar plot of percent of variants classified by region.


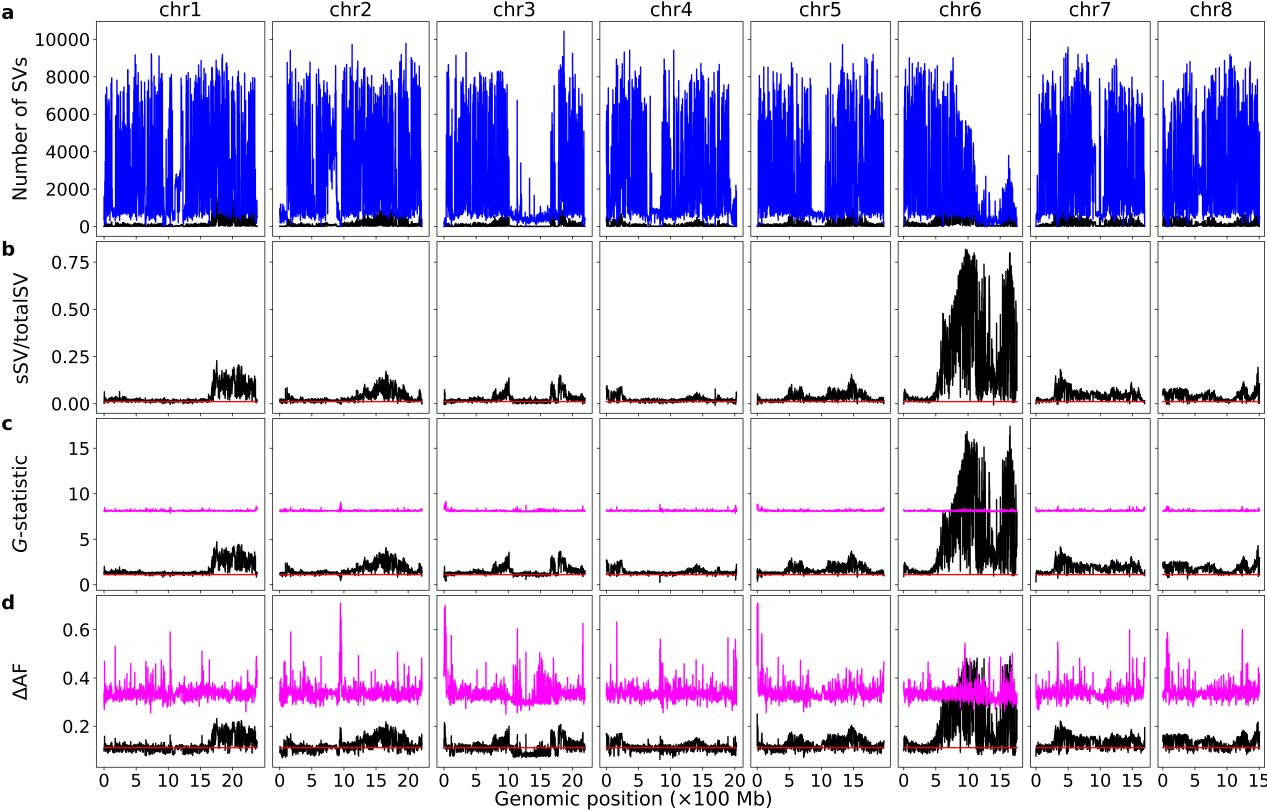


**Figure S2** BSA-Seq data analysis using other methods. The red lines are the thresholds in each method. The blue curves represent total SNPs. The black curves represent the number of sSNPs (a), sSNP/total SNP ratios (b), G-statistic values (c), and ΔAF values (d)


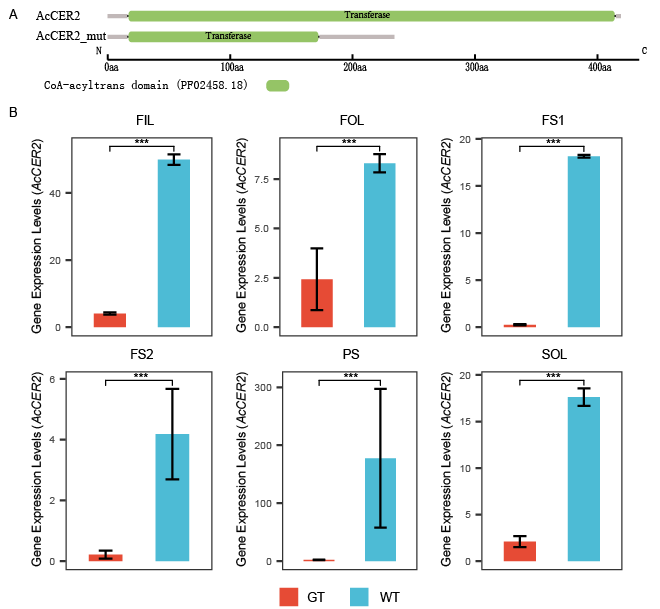


**Figure S3** Structural and functional domains of the *AcCER2* (A), and its expression levels in different tissues and developmental stages in WT and GT accessions (B). The statistical significance of the difference between the GT and WT was confirmed with a wilcox-test. ‘*’ means P value < 0.05, ‘***’ means P value < 0.01, ‘NS’ means P value >=0.05.


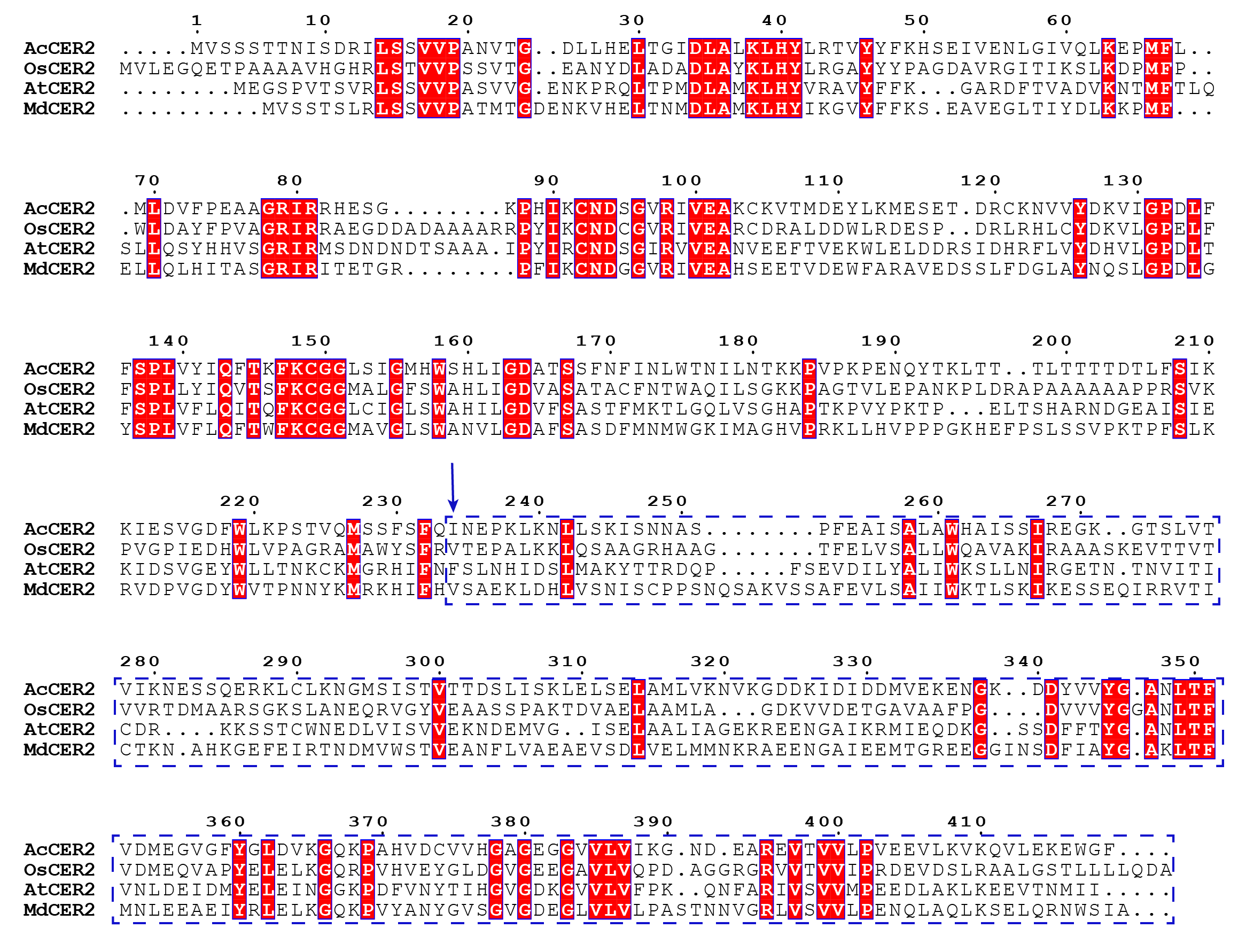


**Figure S4** Multiple sequence alignment of CER2 across different species (onion, rice, *Arabidopsis thaliana*, and apple). Conserved amino acid residues were highlighted in red. The blue arrows indicated the mutation sites in AcCER2, which result in the loss of the amino acid sequence enclosed within the blue dashed box.


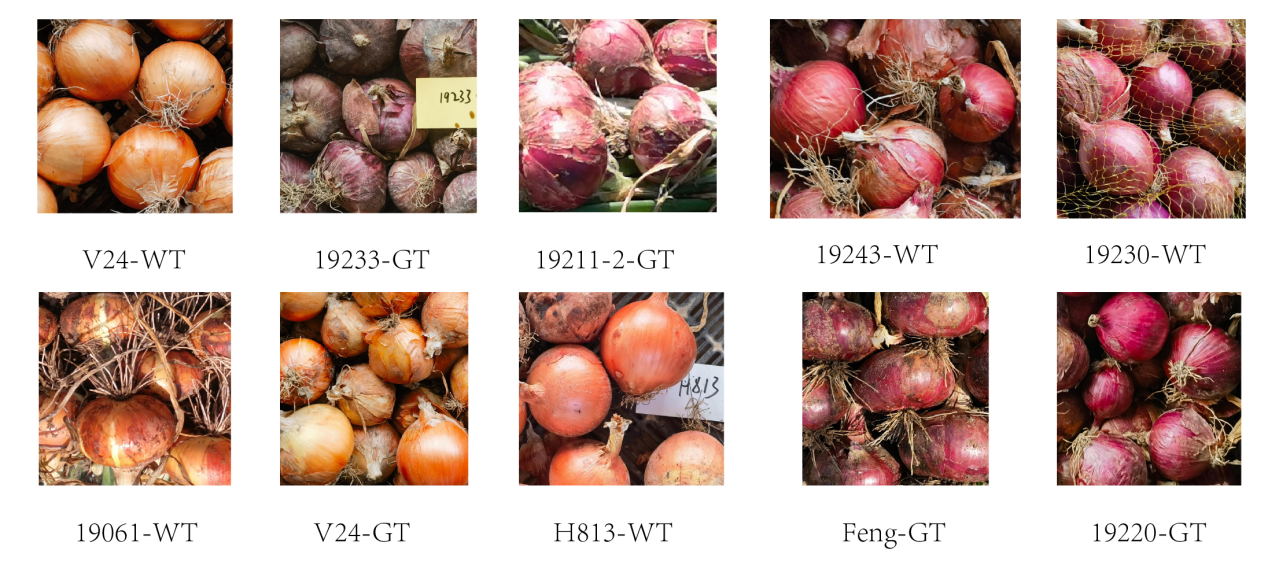


**Figure S5** Different phenotypes of onion accessions used for validation.


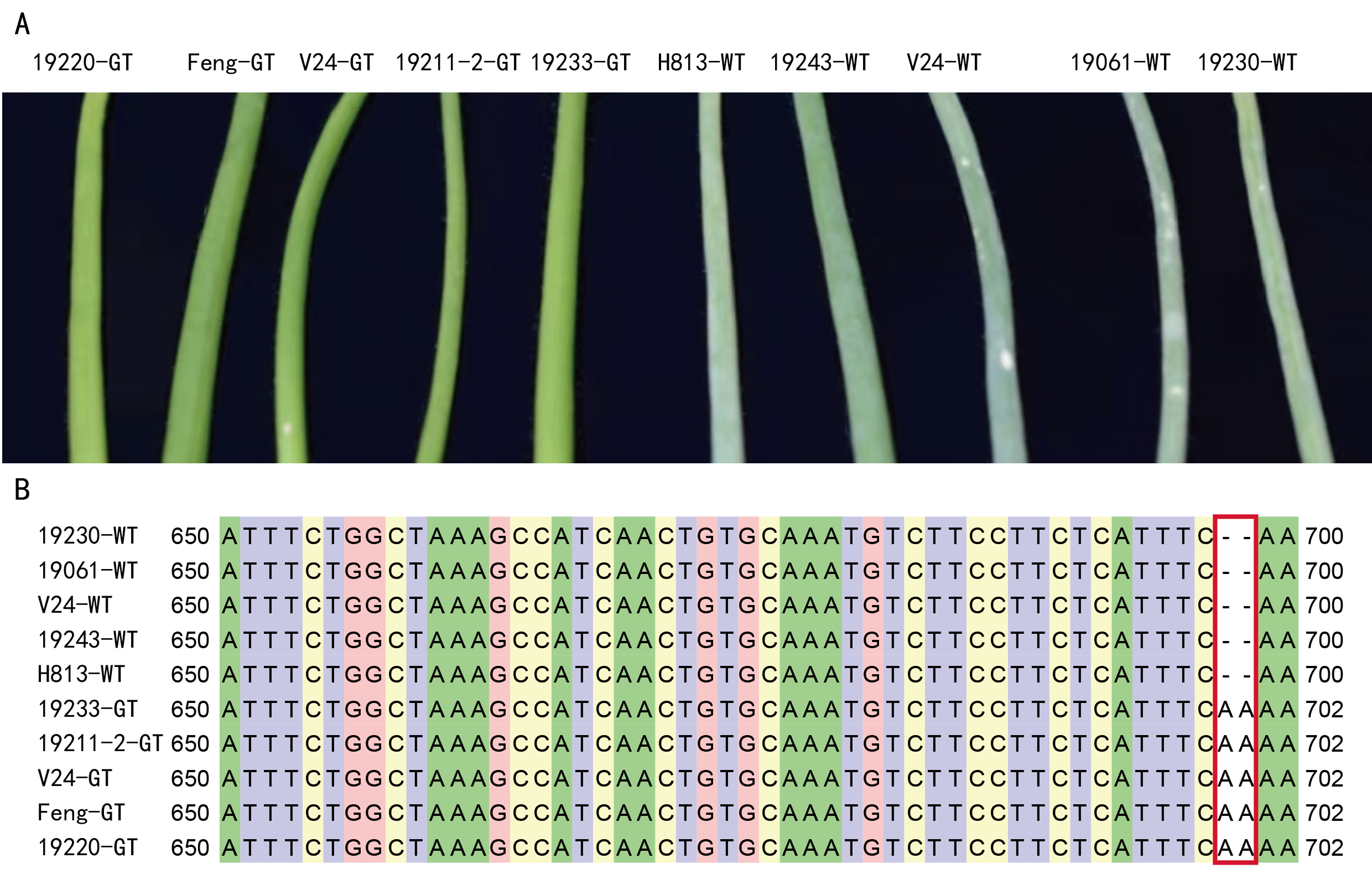


**Figure S6** Leaf phenotypes of onion accessions used for validation (A) and sequences near the *AcCER2* mutation site in these onion accessions (B).


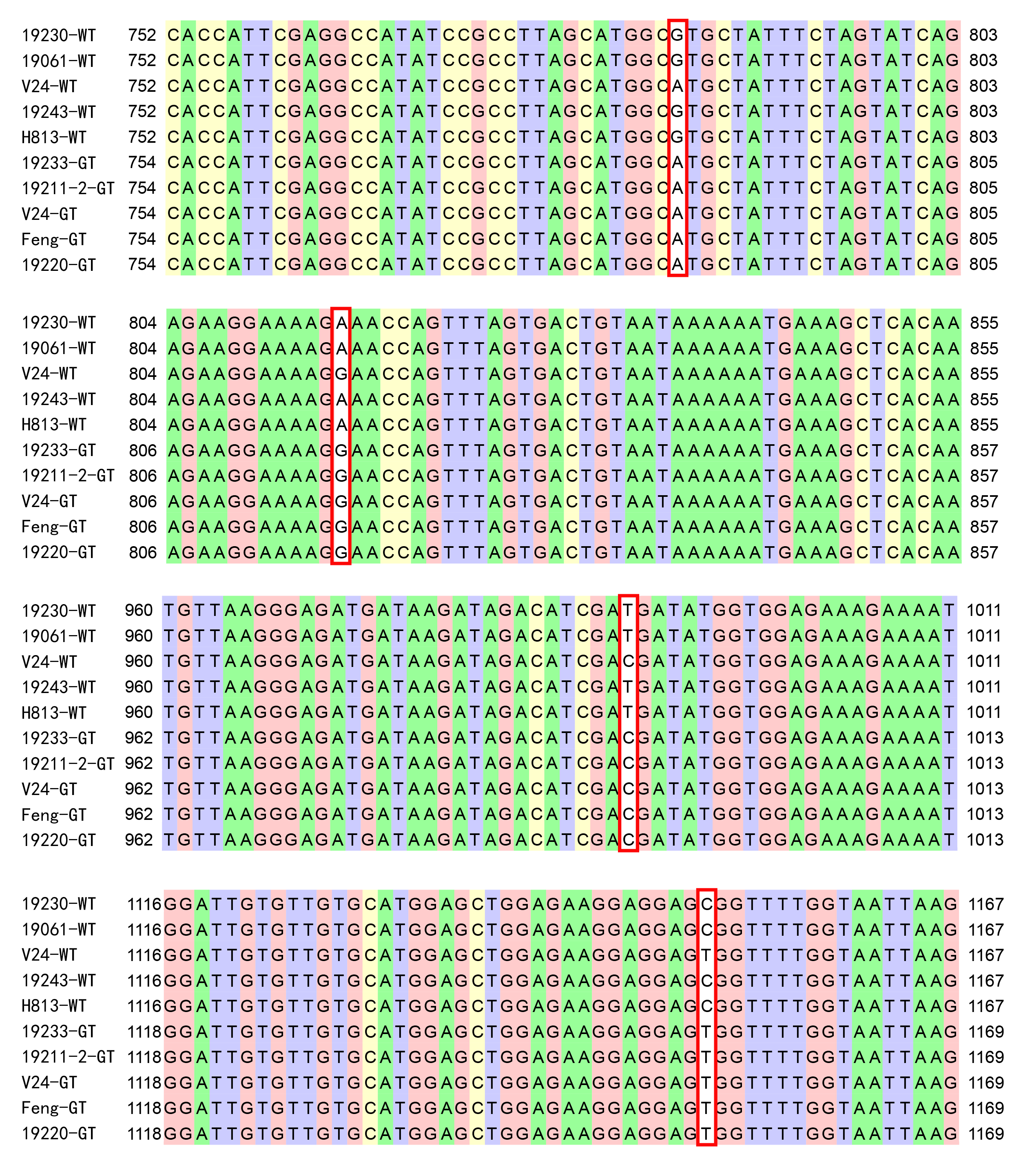


**Figure S7** Polymorphic sites in the *AcCER2* gene of different onion accessions.


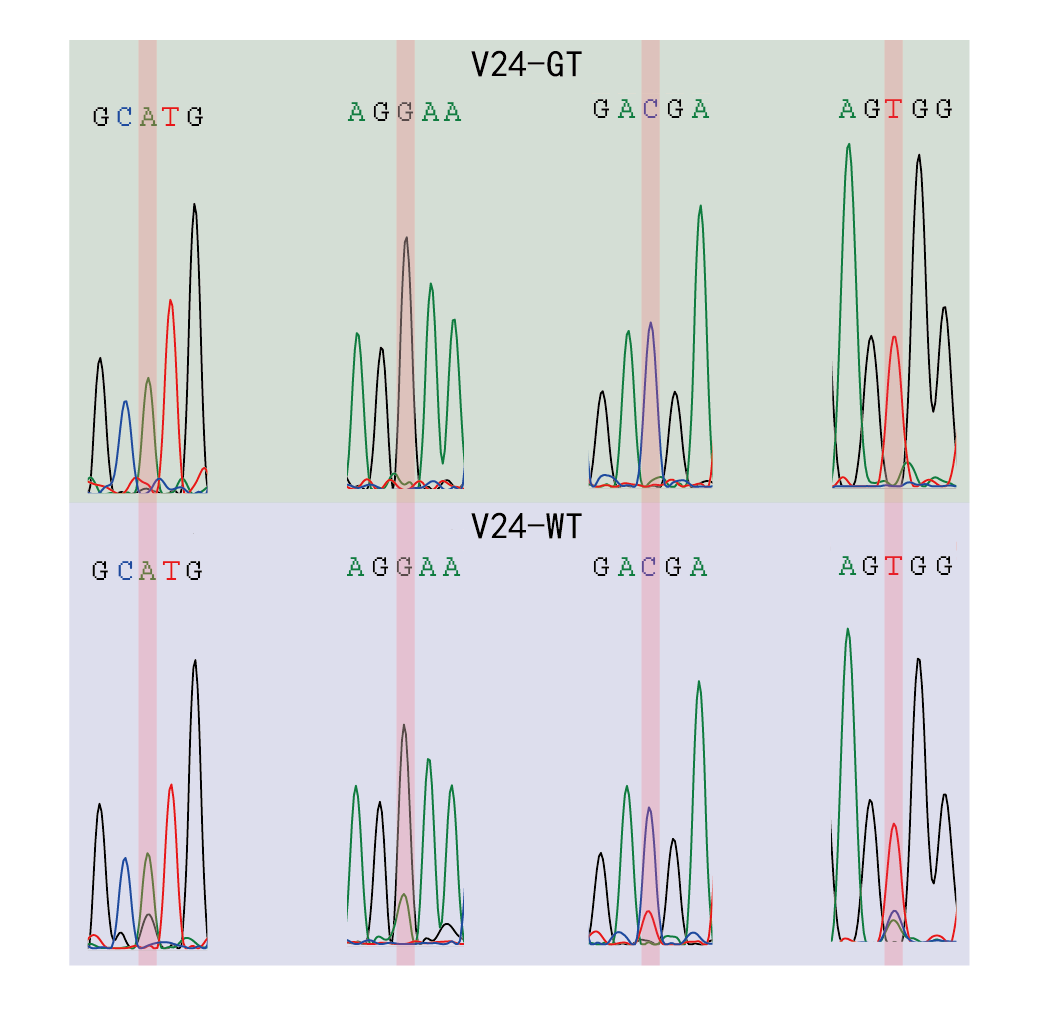


**Figure S8** Sanger sequencing chromatograms of polymorphic sites in the V24-GT and V24-WT accessions. The presence of two peaks at the same site typically indicates heterozygosity at that position


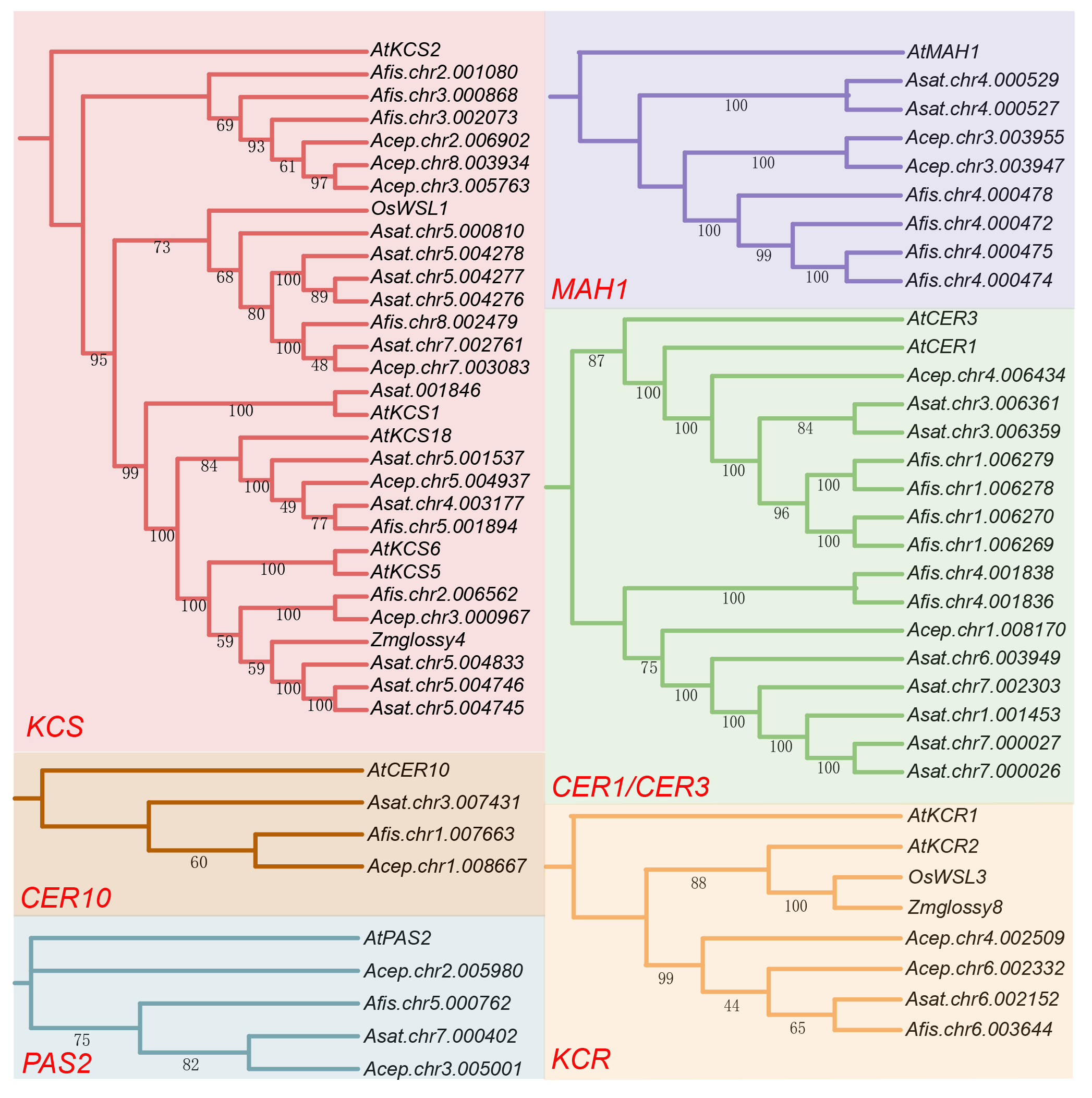


**Figure S9** Phylogenetic analysis of genes encoding key enzymes for the biosynthesis of 16-hentriacontanone in the onion, rice, maize, and *Arabidopsis thaliana*


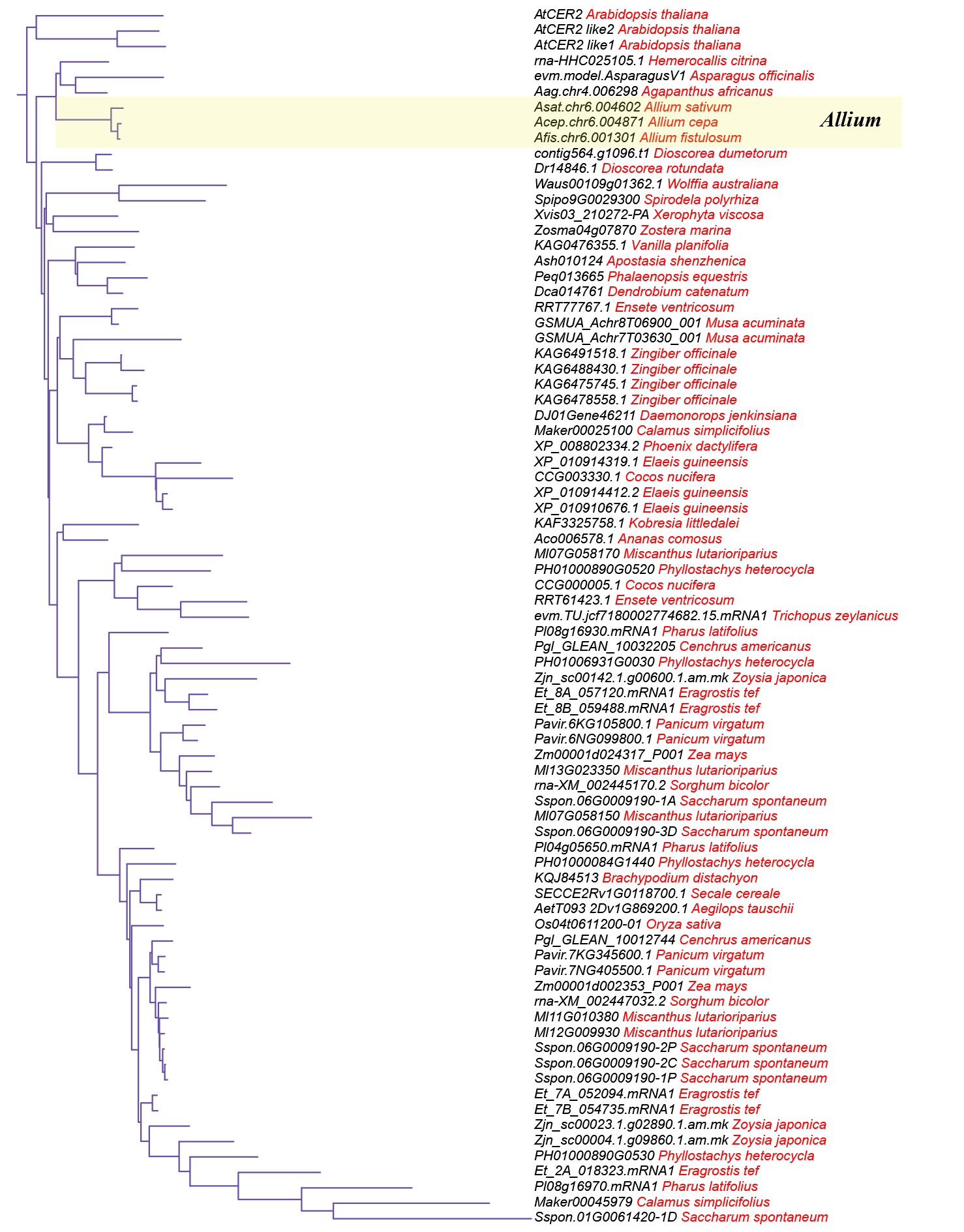


**Figure S10** Phylogenetic analysis of *CER2* genes in monocot plants and *Arabidopsis thaliana*.

**
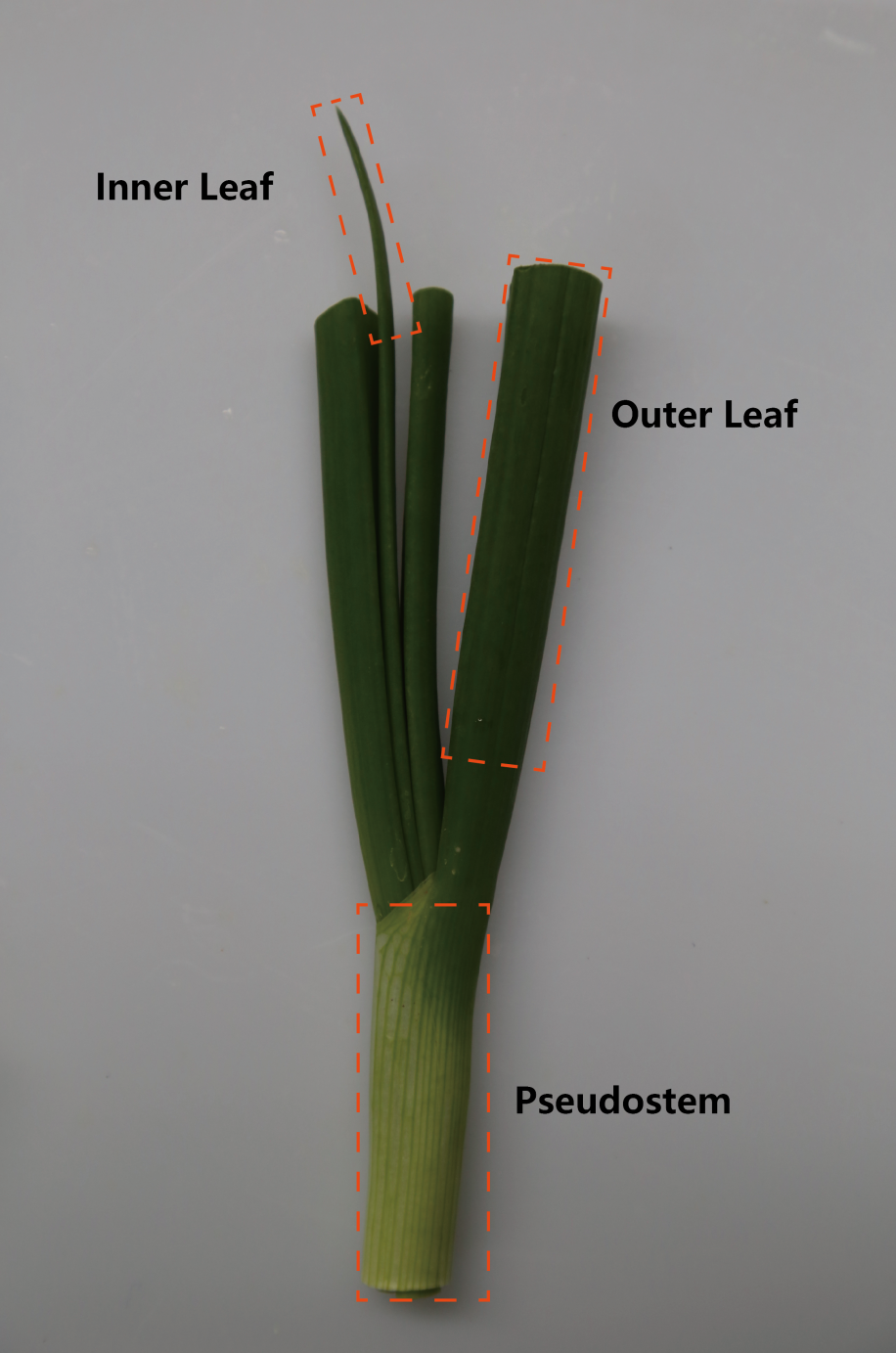
**

**Figure S11** Marked collection sites of onion tissues for RNA-seq analysis.


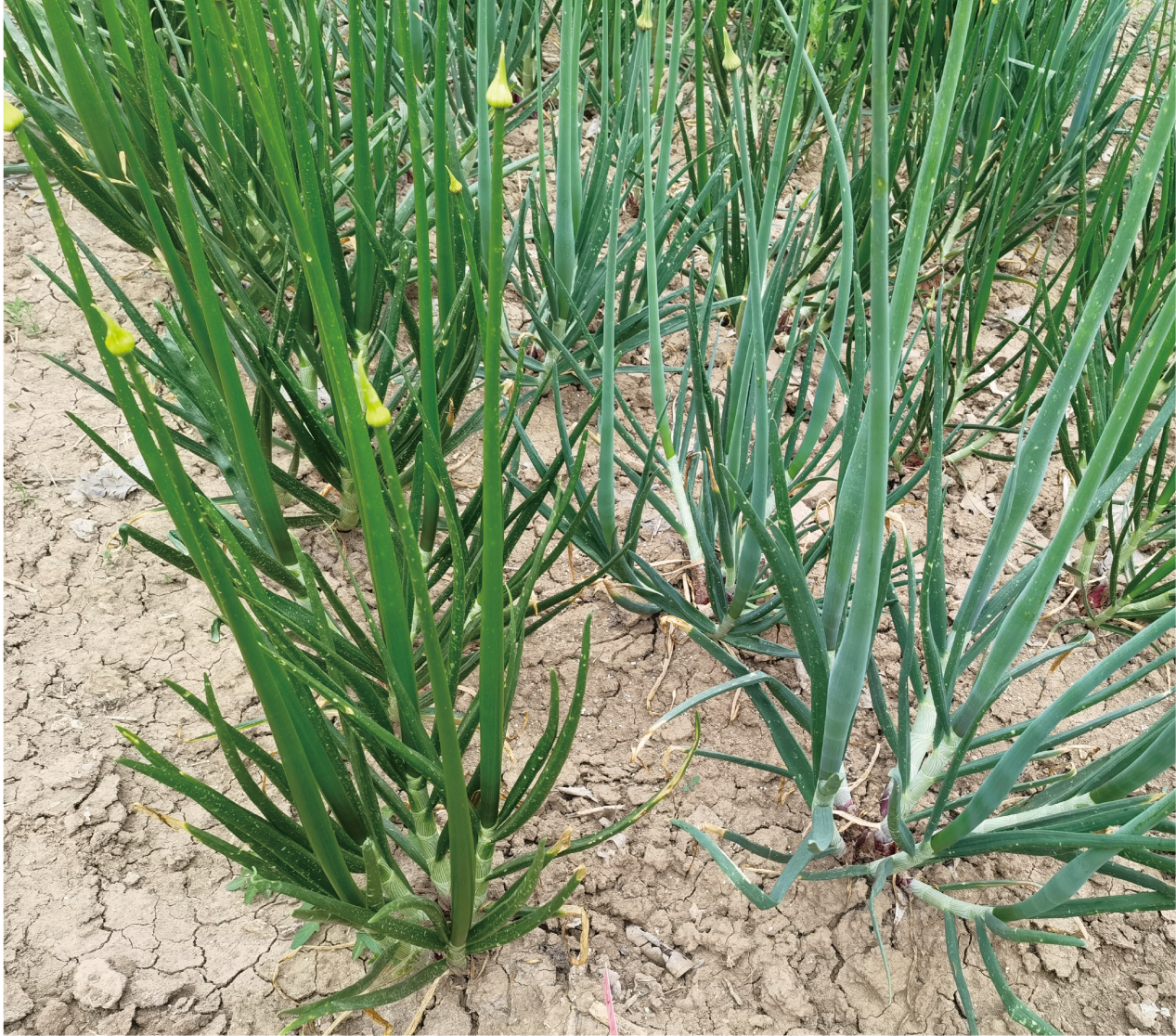


**Figure S12** Field phenotypes of GT (right) and WT (left) onions.


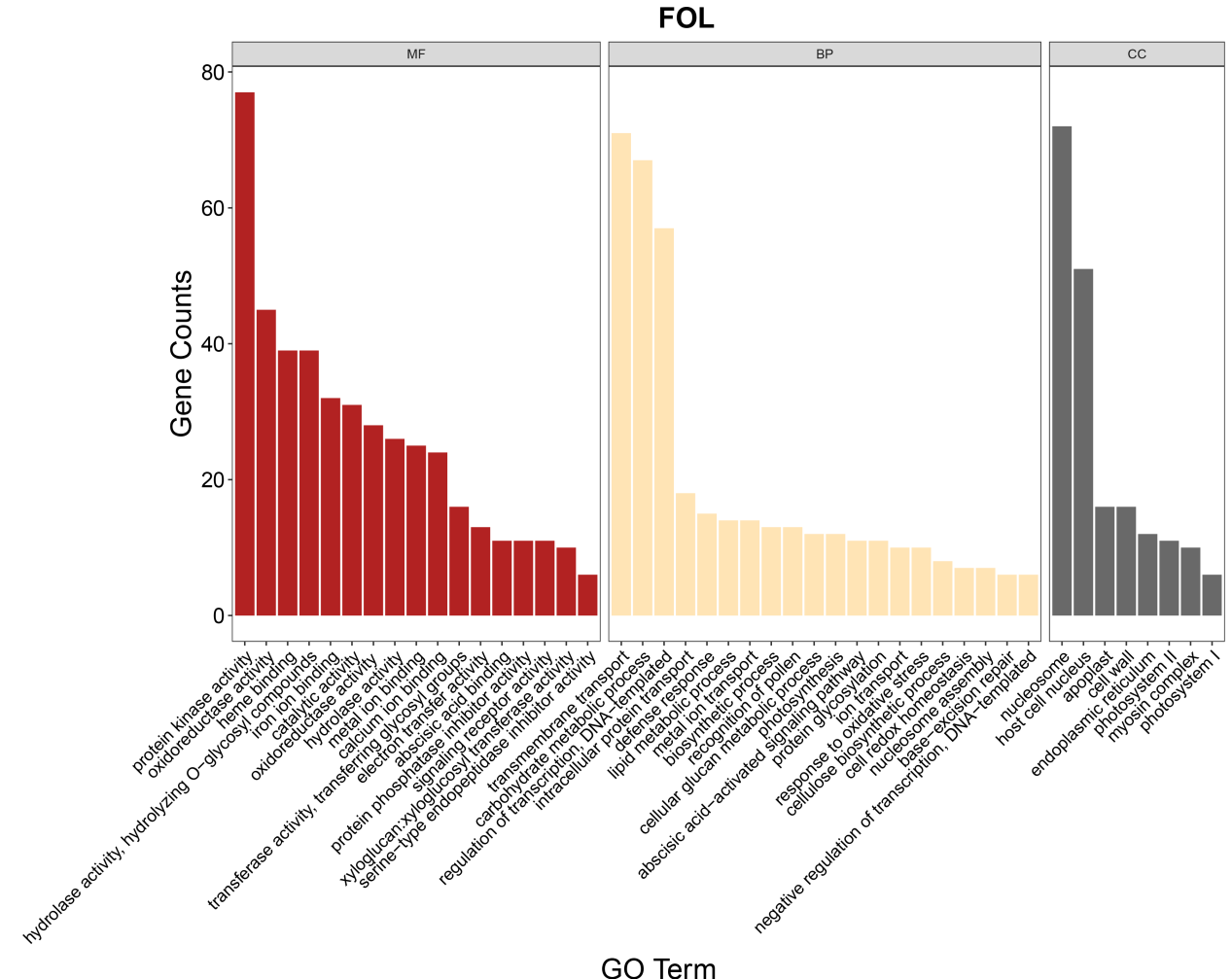


**Figure S13** The bar plot of significant GO terms enriched in DEGs in FOL. Red bars represent terms related to ‘Molecular Functions’, yellow bars denote terms associated with ‘Biological Processes’, while gray bars represent terms related to ‘Cell Components’.


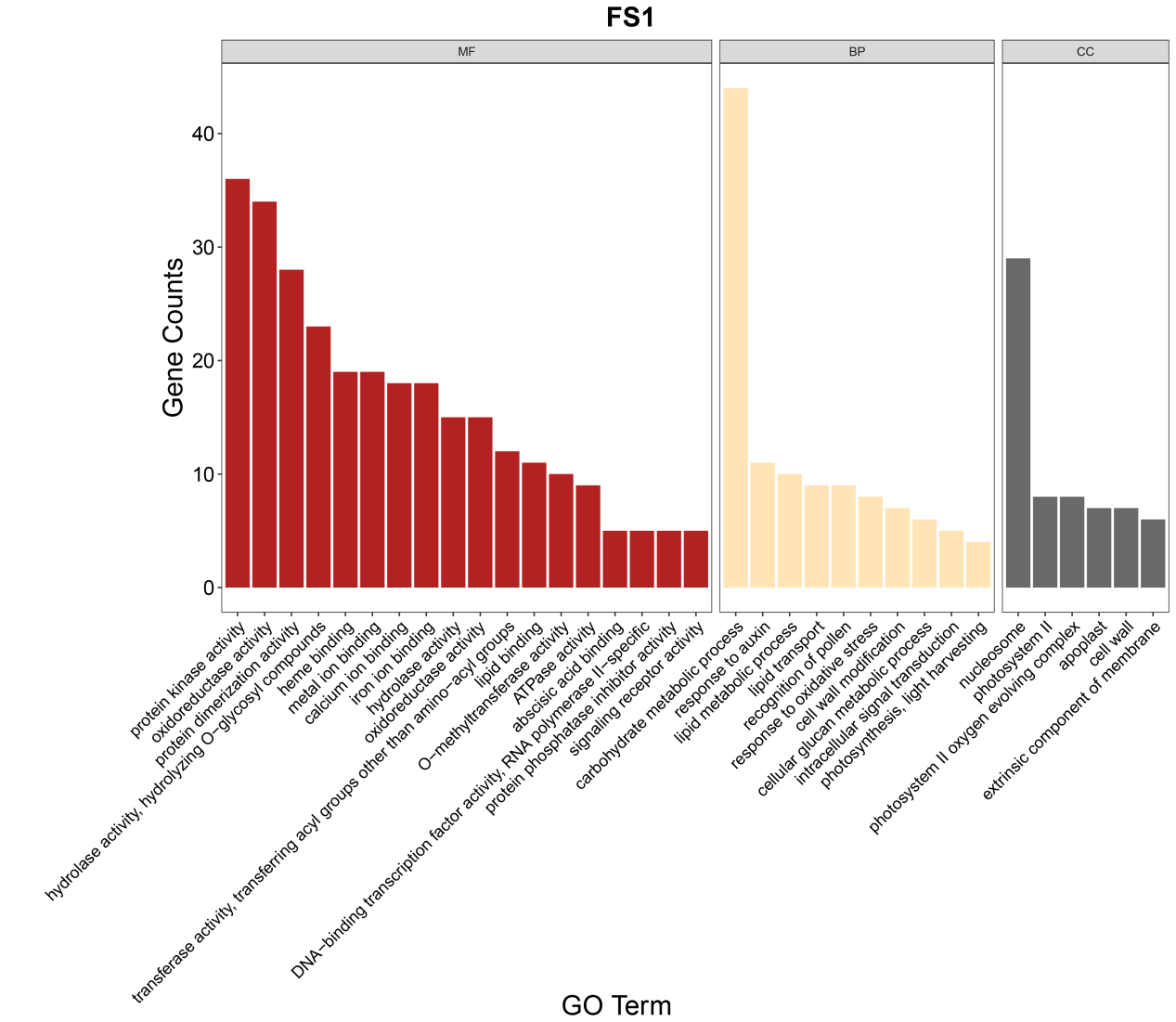


**Figure S14** The bar plot of significant GO terms enriched in DEGs in FS1. Red bars represent terms related to ‘Molecular Functions’, yellow bars denote terms associated with ‘Biological Processes’, while gray bars represent terms related to ‘Cell Components’.


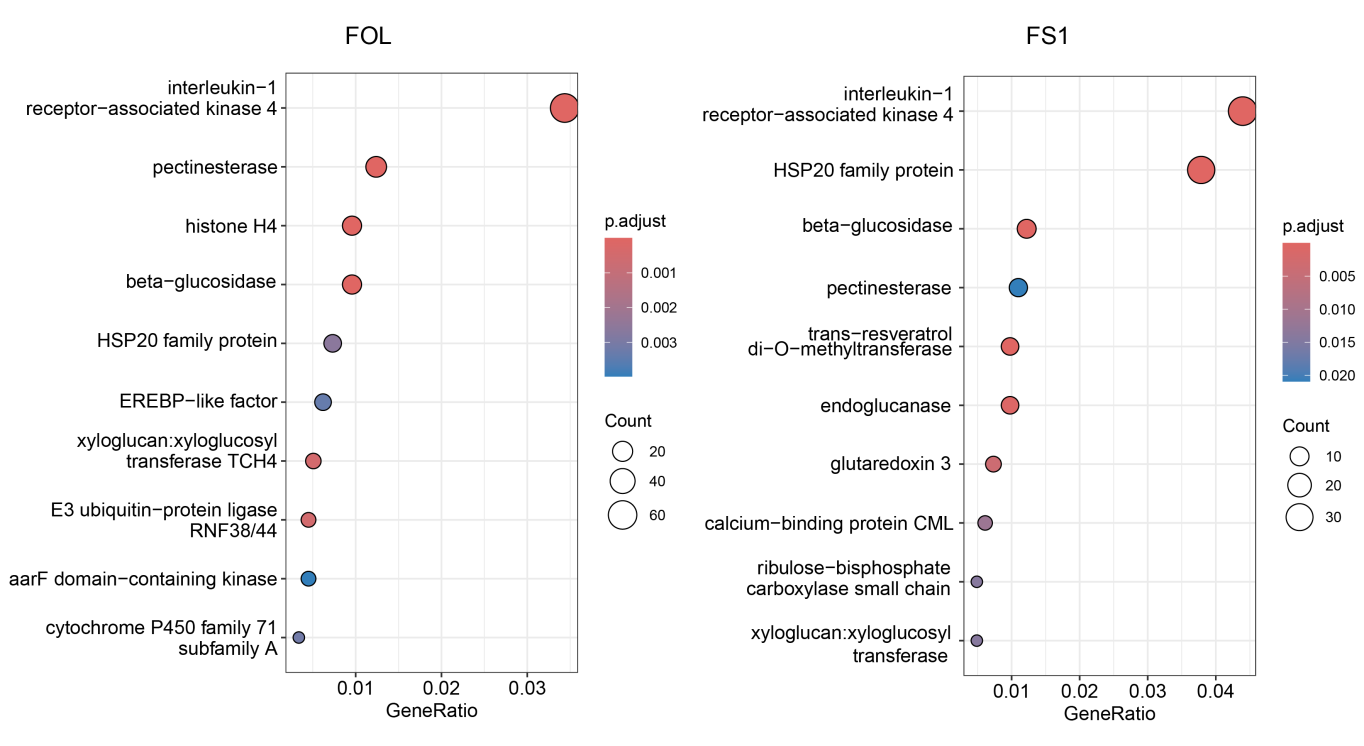


**Figure S15** The bubble plot of significant KO terms enriched in DEGs in FOL (left) and FS1(right). Each bubble represents a KO term, with its size indicating the gene counts and the color denoting the P-value, reflecting the statistical significance of the enrichment.


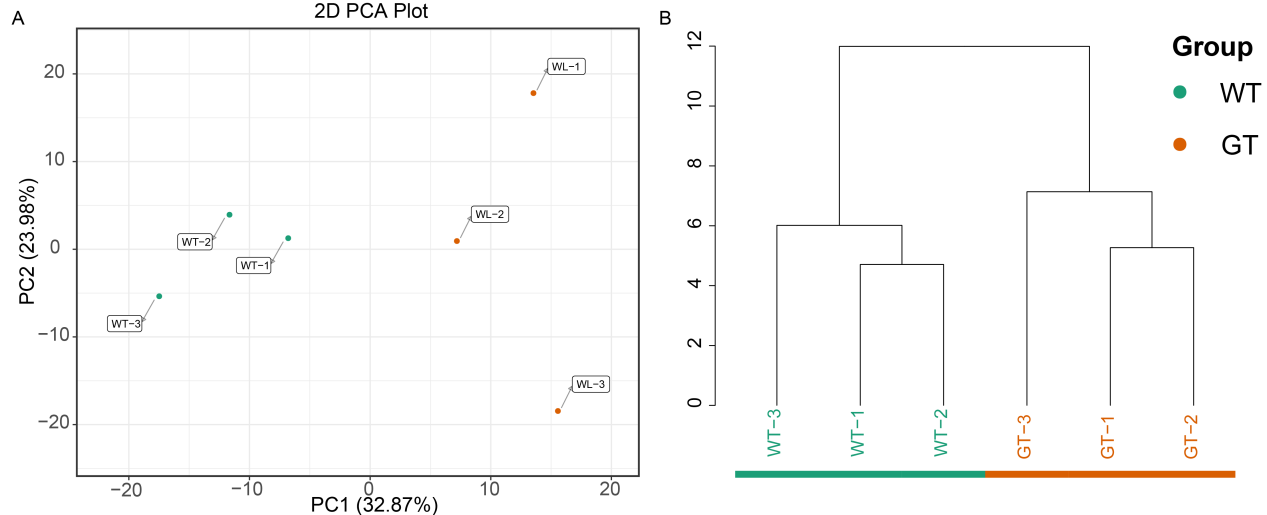


**Figure S16** (A) Principal component analysis (PCA) scatter plot of metabolites, which displays the separation between GT and WT accessions, with the X-axis representing PC1 and the Y-axis representing PC2. (B) Metabolite-based cluster analysis of GT and WT accessions


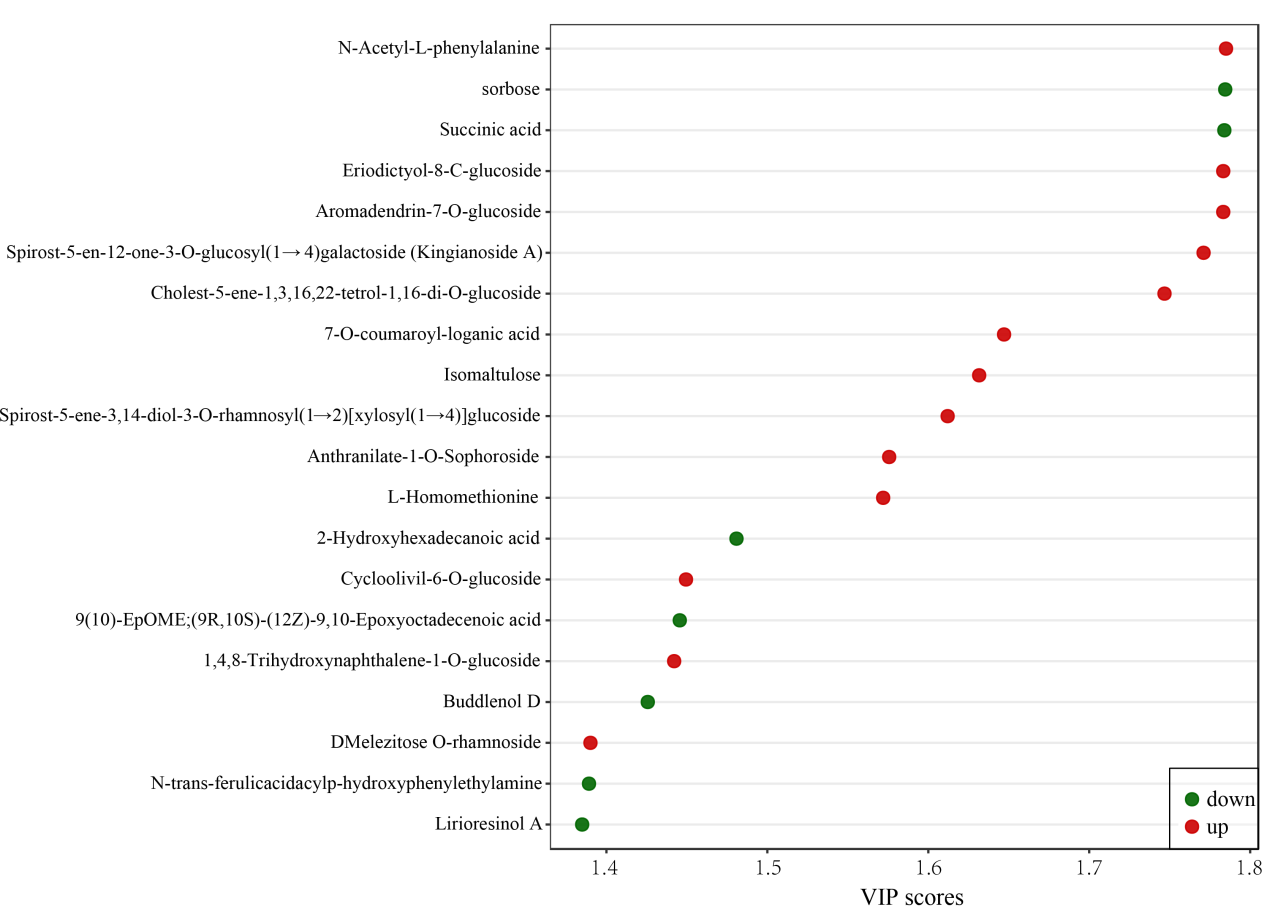


**Figure S17** The dot plot of the max VIP scores of metabolites (TOP 20)
