# Supplementary figures and images for: A premature termination codon mutation in the onion AcCER2 gene is associated with both glossy leaves and thrip resistance
Source: Hortic Res. 2025 Jan 14;12(4):uhaf006. doi: 10.1093/hr/uhaf006 (PMC11896967; doi:10.1093/hr/uhaf006)

Percent of variants by region

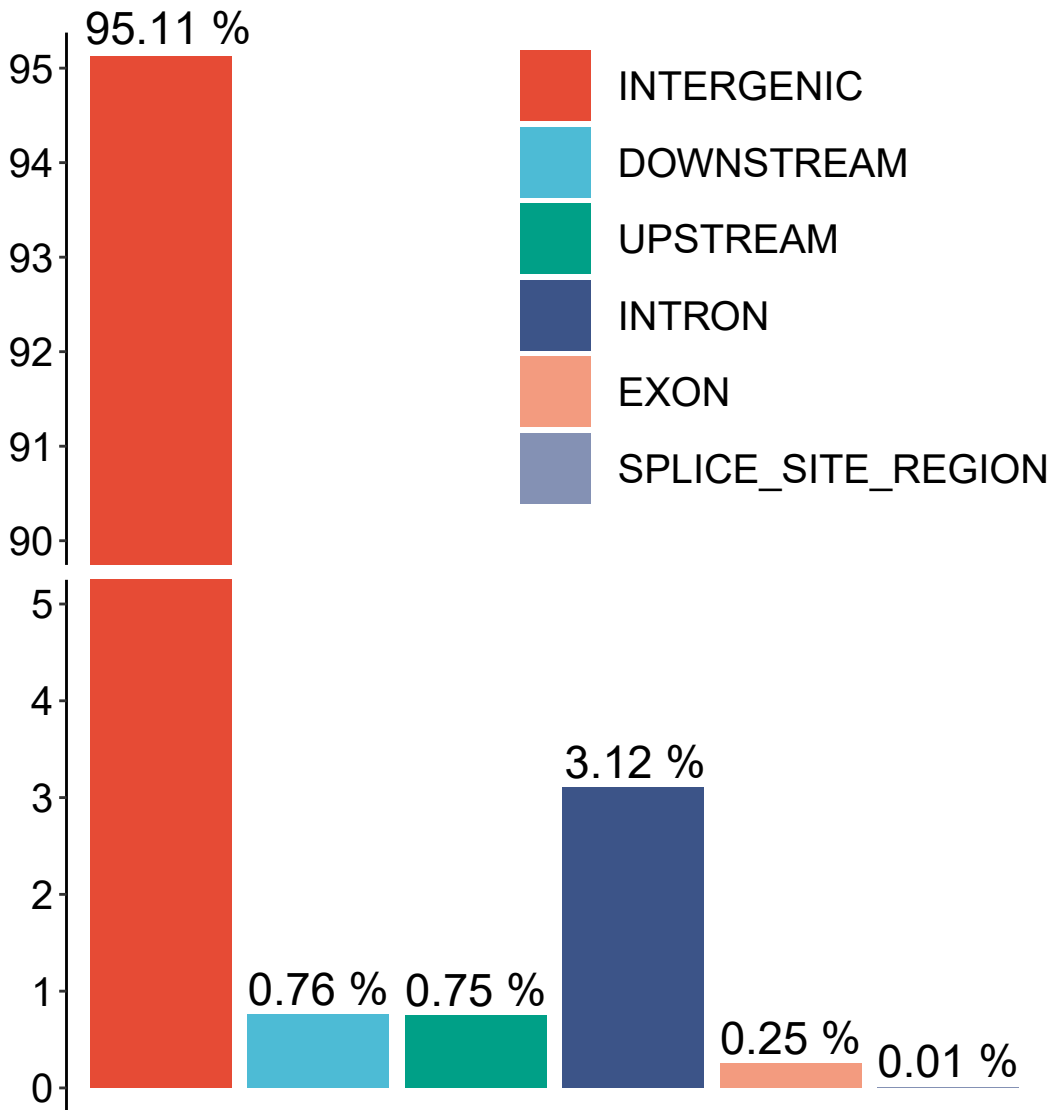

Supplement: Web_Material_uhaf006 [file web_material_uhaf006.zip › Figure S1.pdf]

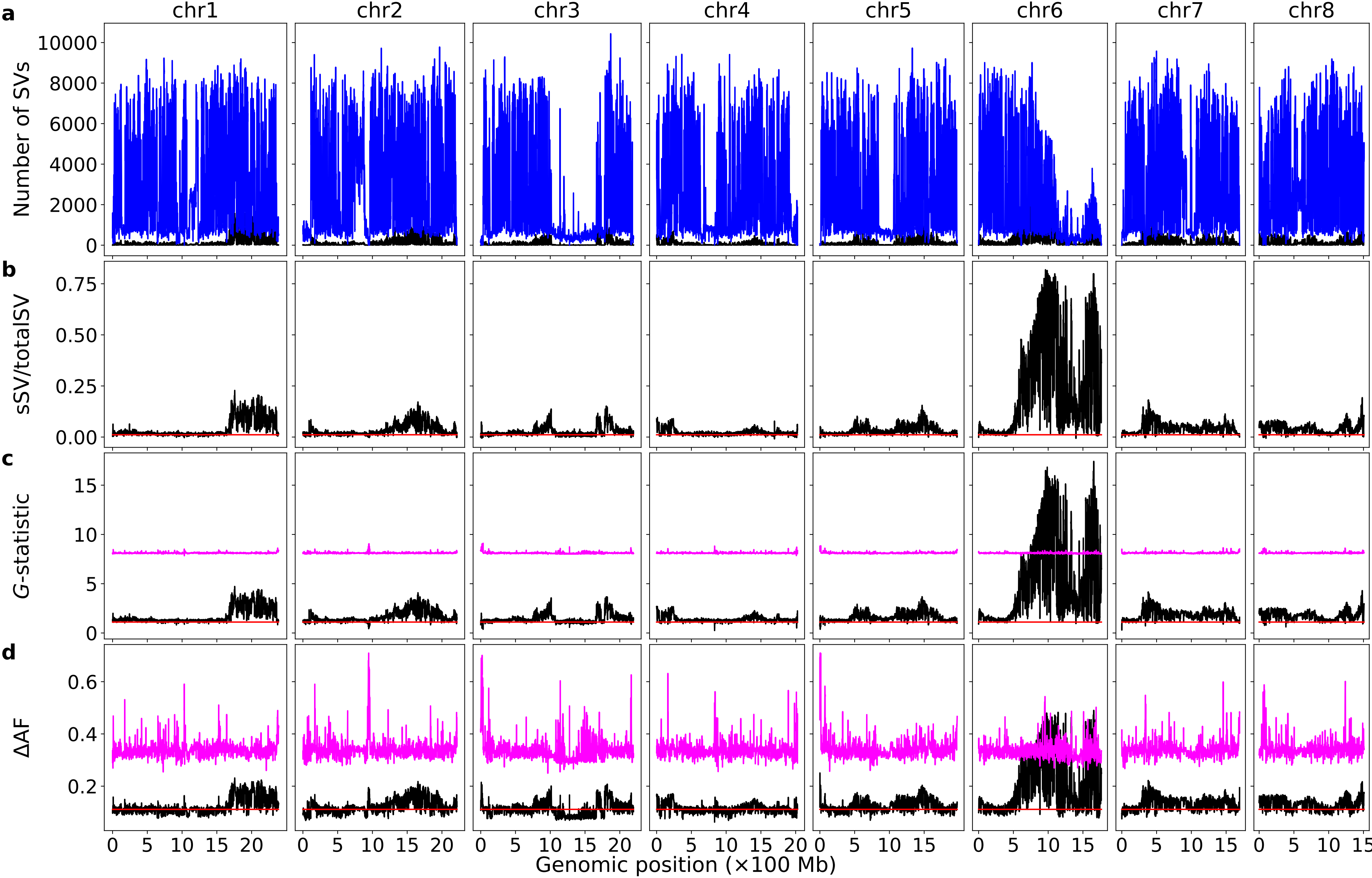

Supplement: Web_Material_uhaf006 [file web_material_uhaf006.zip › Figure S2.pdf]

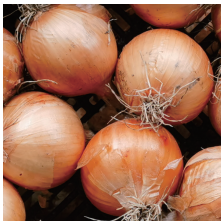

V24-WT

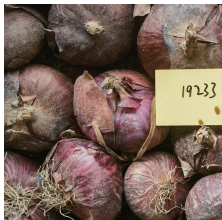

19233-GT

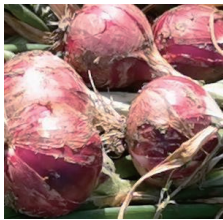

19211-2-GT

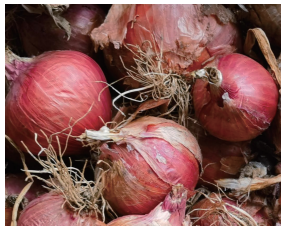

19243-WT

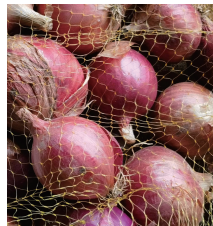

19230-WT

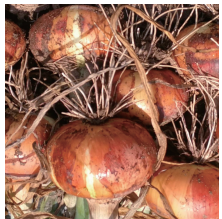

19061-WT

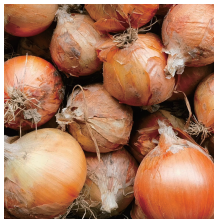

V24-GT

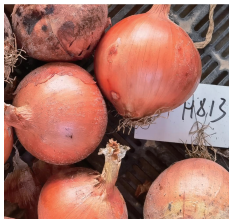

H813-WT

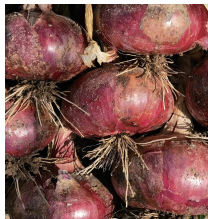

Feng-GT

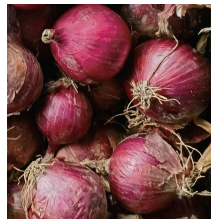

19220-GT

Supplement: Web_Material_uhaf006 [file web_material_uhaf006.zip › Figure S5.pdf]

# V24-GT

GCATG

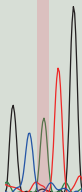

AGGAA

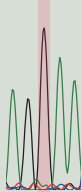

GACGA

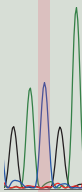

AGTGG

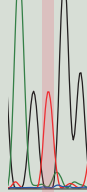

# V24-WT

GCATG

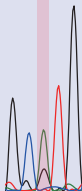

AGGAA

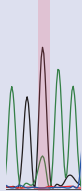

GACGA

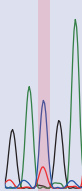

AGTGG

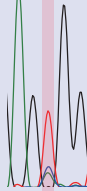

Supplement: Web_Material_uhaf006 [file web_material_uhaf006.zip › Figure S8.pdf]

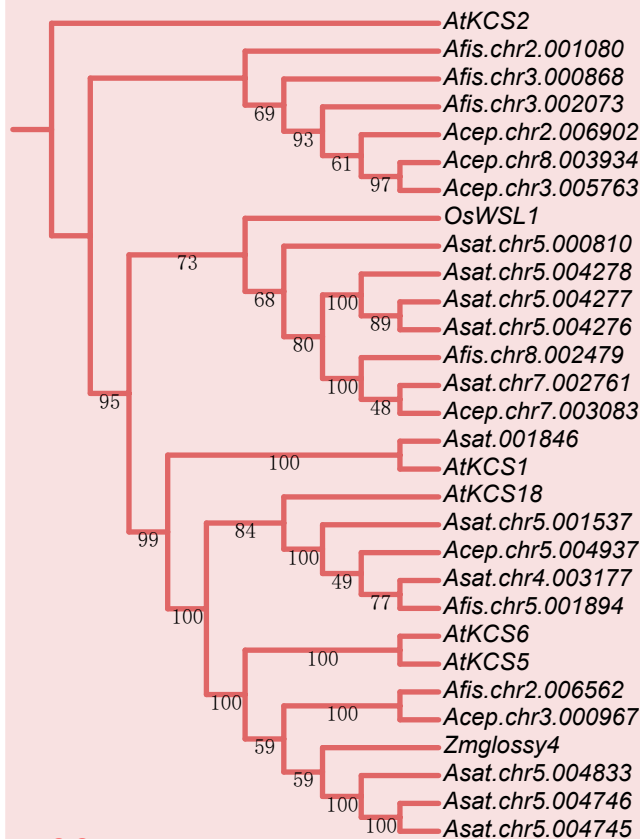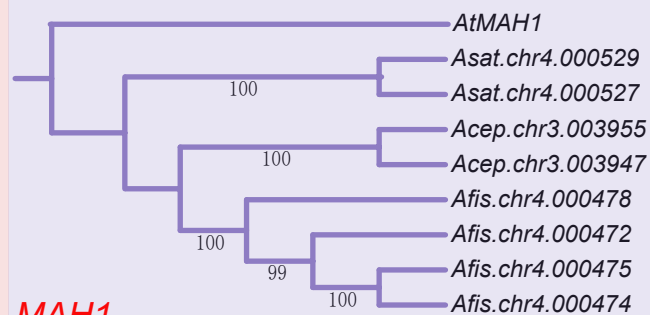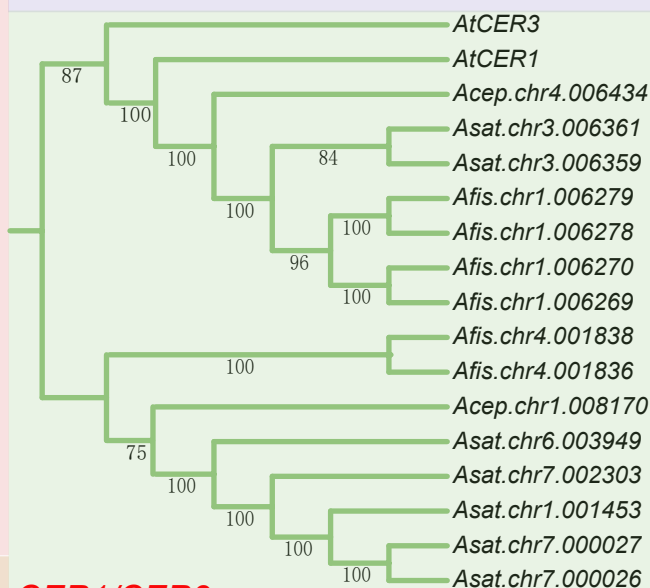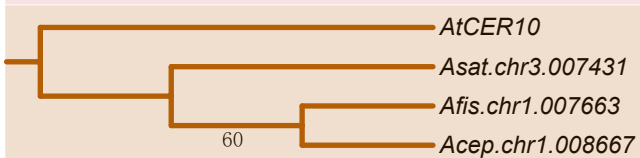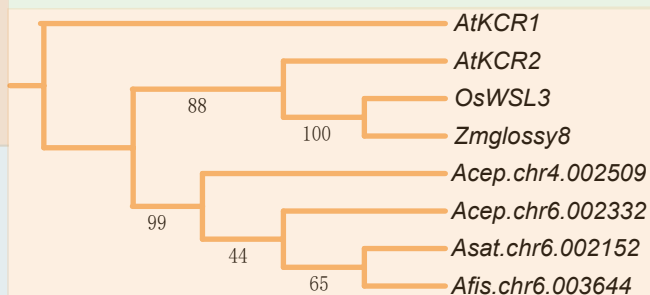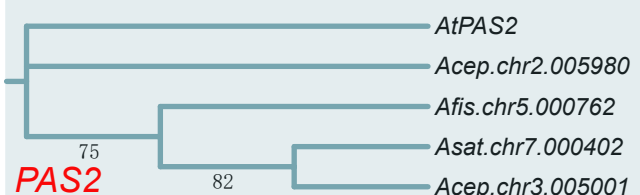

Supplement: Web_Material_uhaf006 [file web_material_uhaf006.zip › Figure S9.pdf]

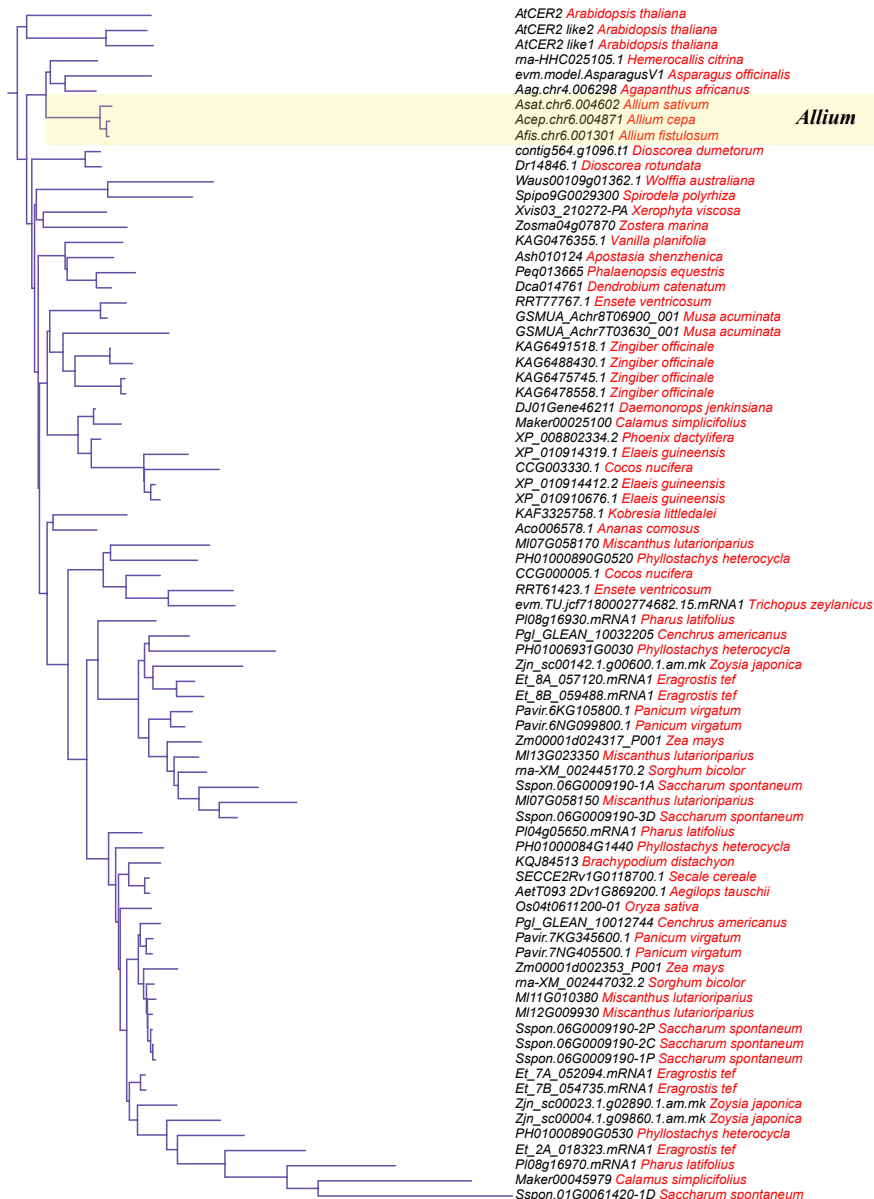

Supplement: Web_Material_uhaf006 [file web_material_uhaf006.zip › Figure S10.pdf]

**Inner Leaf**

**Outer Leaf**

**Pseudostem**

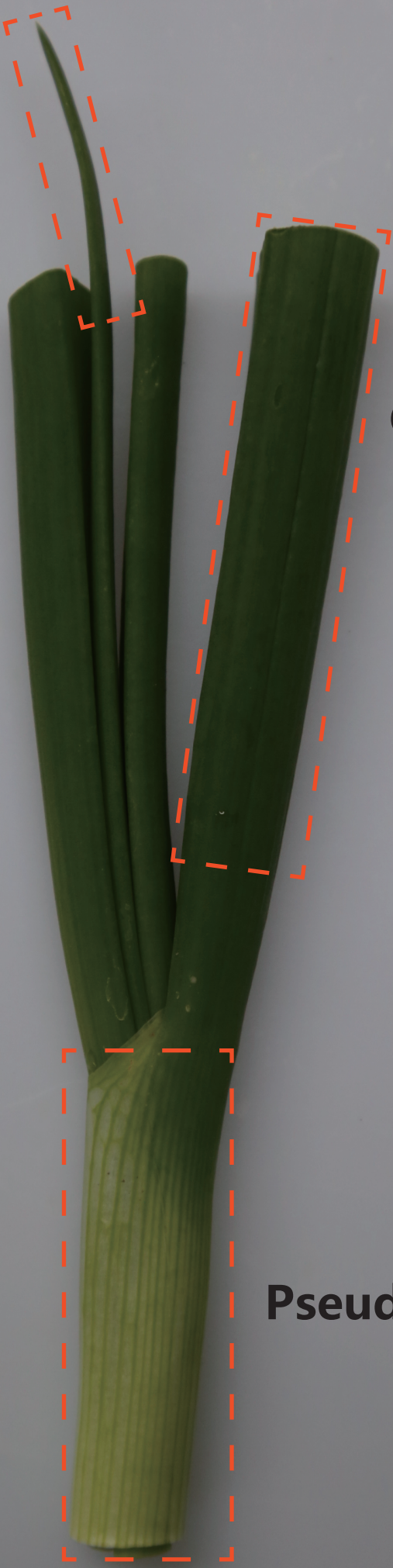

Supplement: Web_Material_uhaf006 [file web_material_uhaf006.zip › Figure S11.pdf]

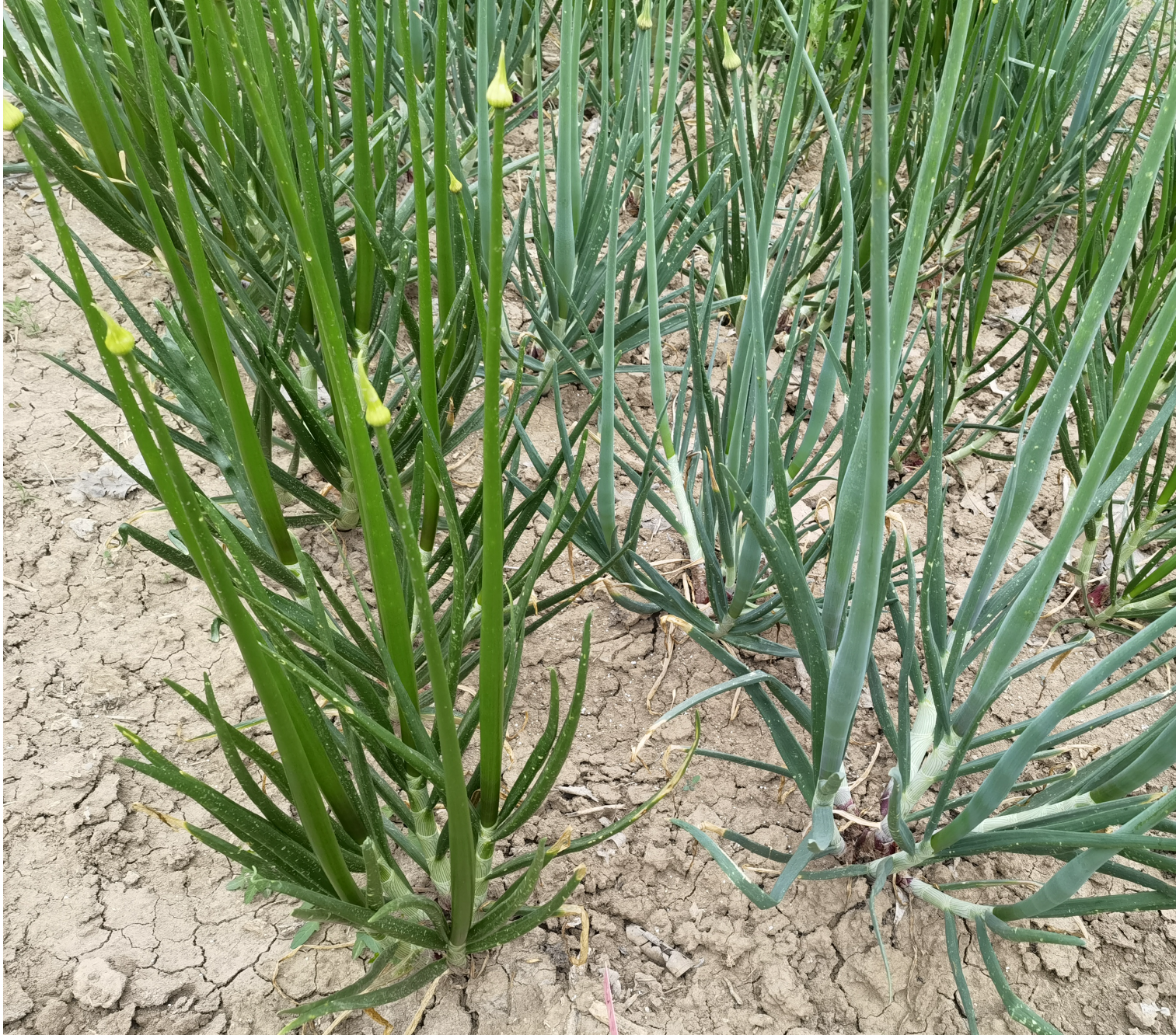

Supplement: Web_Material_uhaf006 [file web_material_uhaf006.zip › Figure S12.pdf]

# FOL

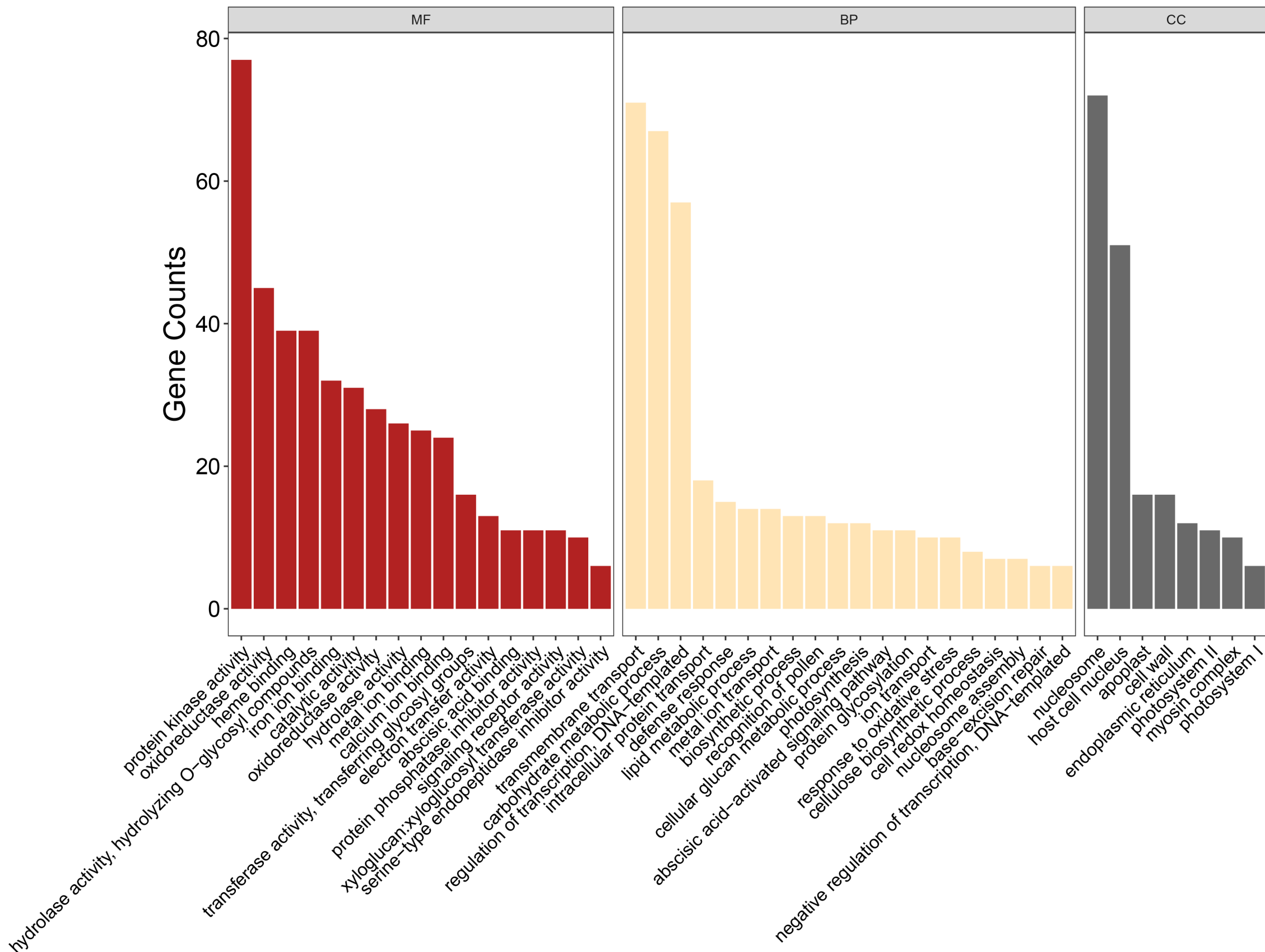

GO Term

# FOL

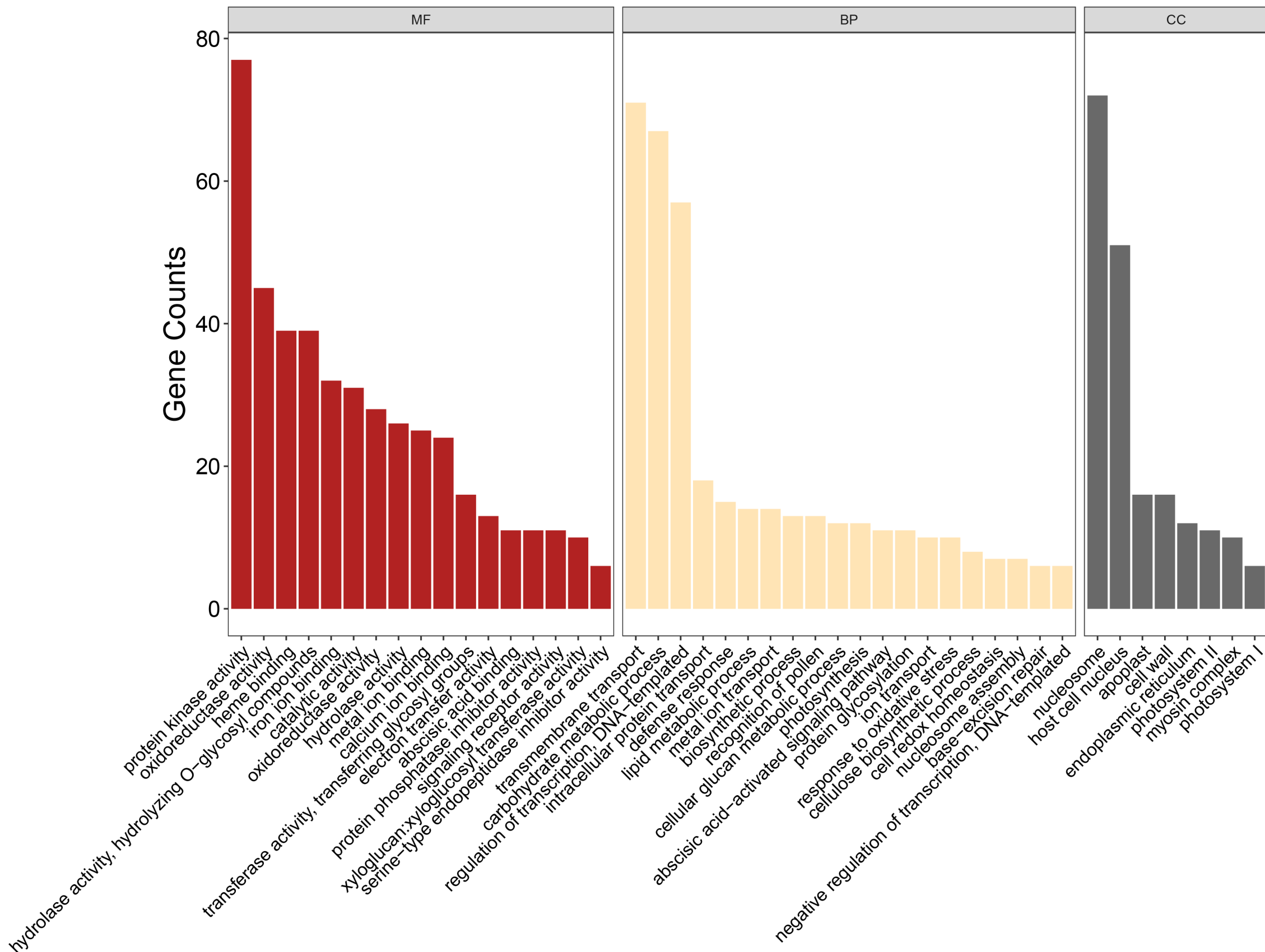

GO Term

Supplement: Web_Material_uhaf006 [file web_material_uhaf006.zip › Figure S13.pdf]

# FS1

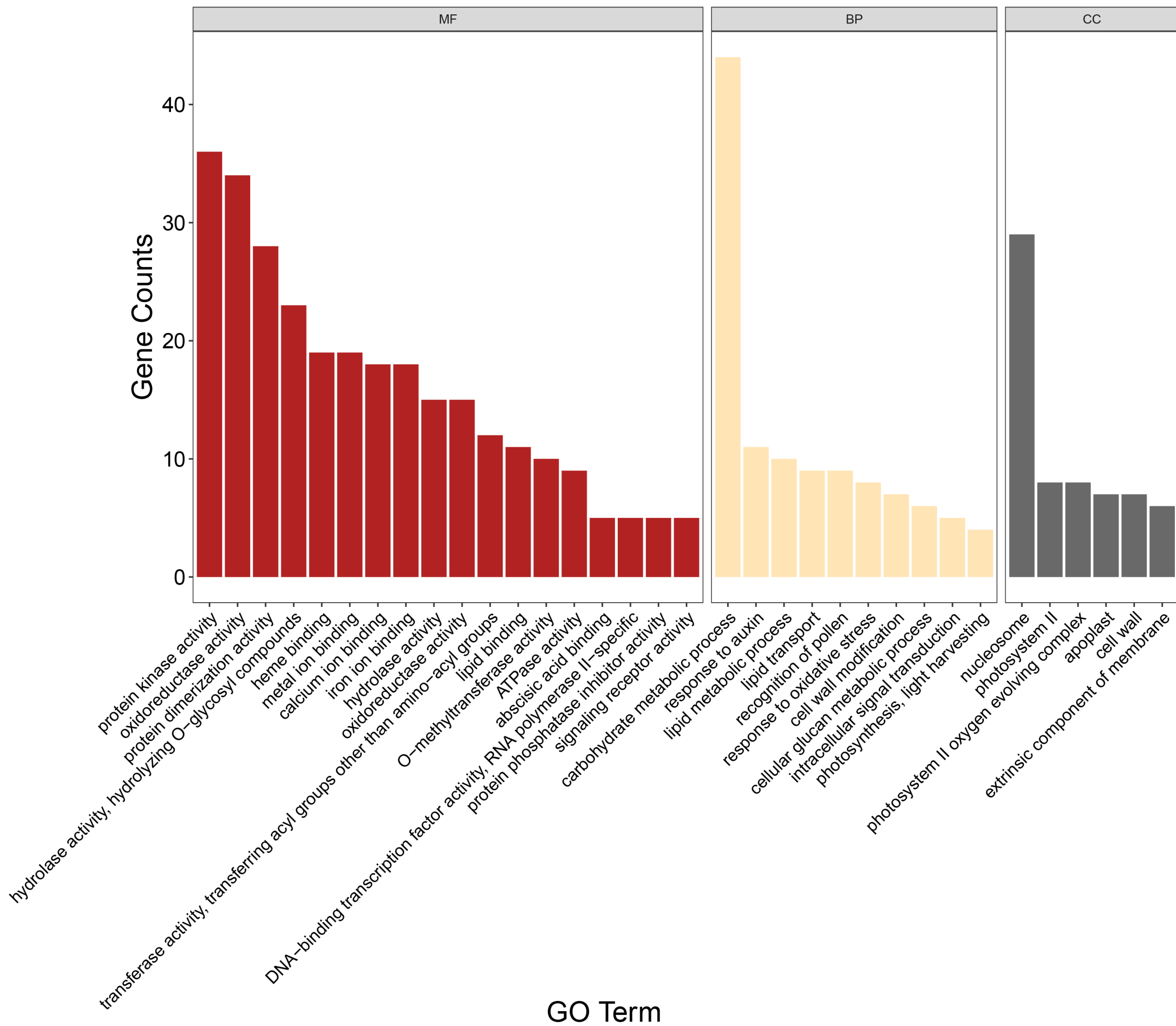

Supplement: Web_Material_uhaf006 [file web_material_uhaf006.zip › Figure S14.pdf]

FOL

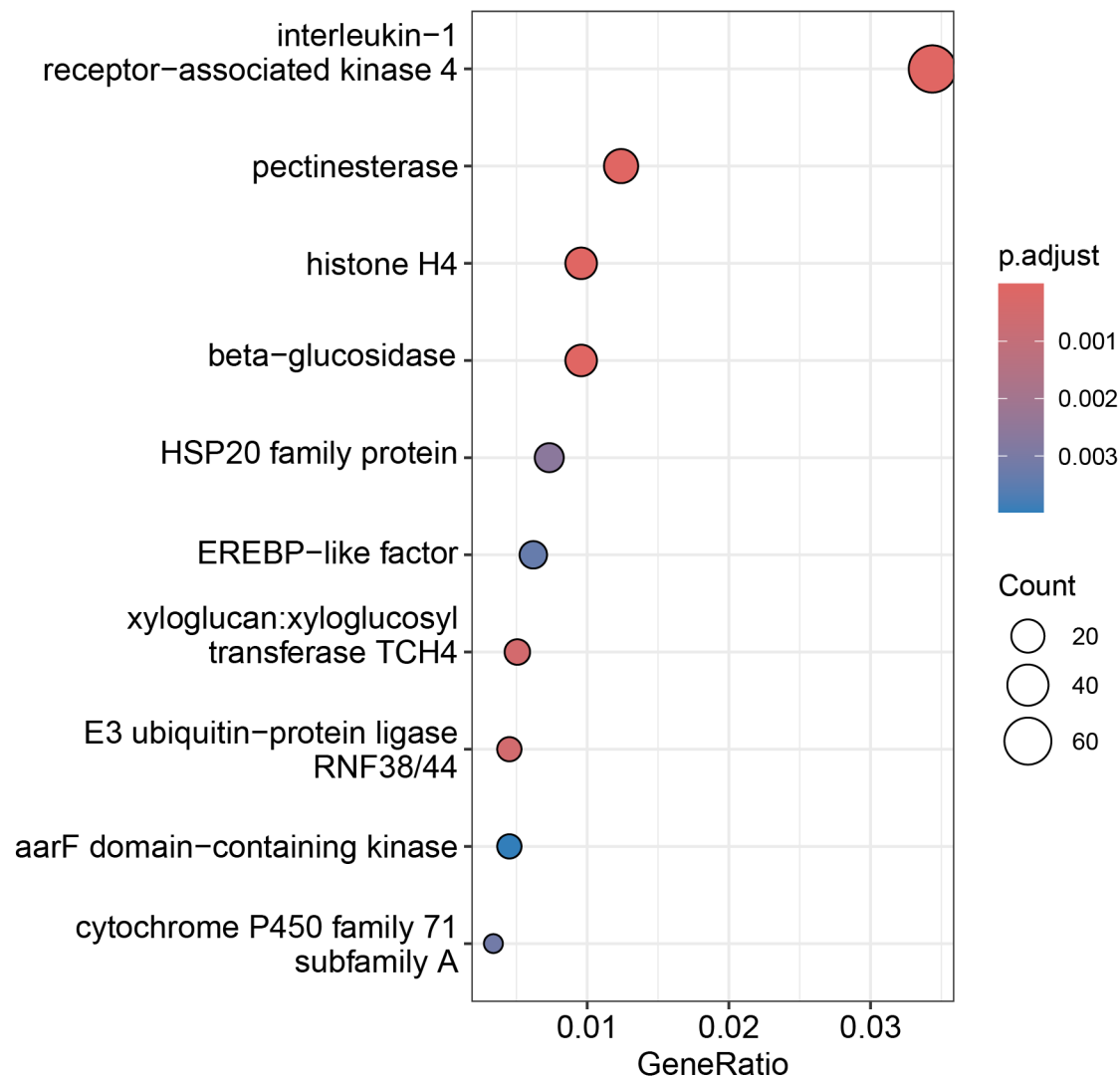

FS1

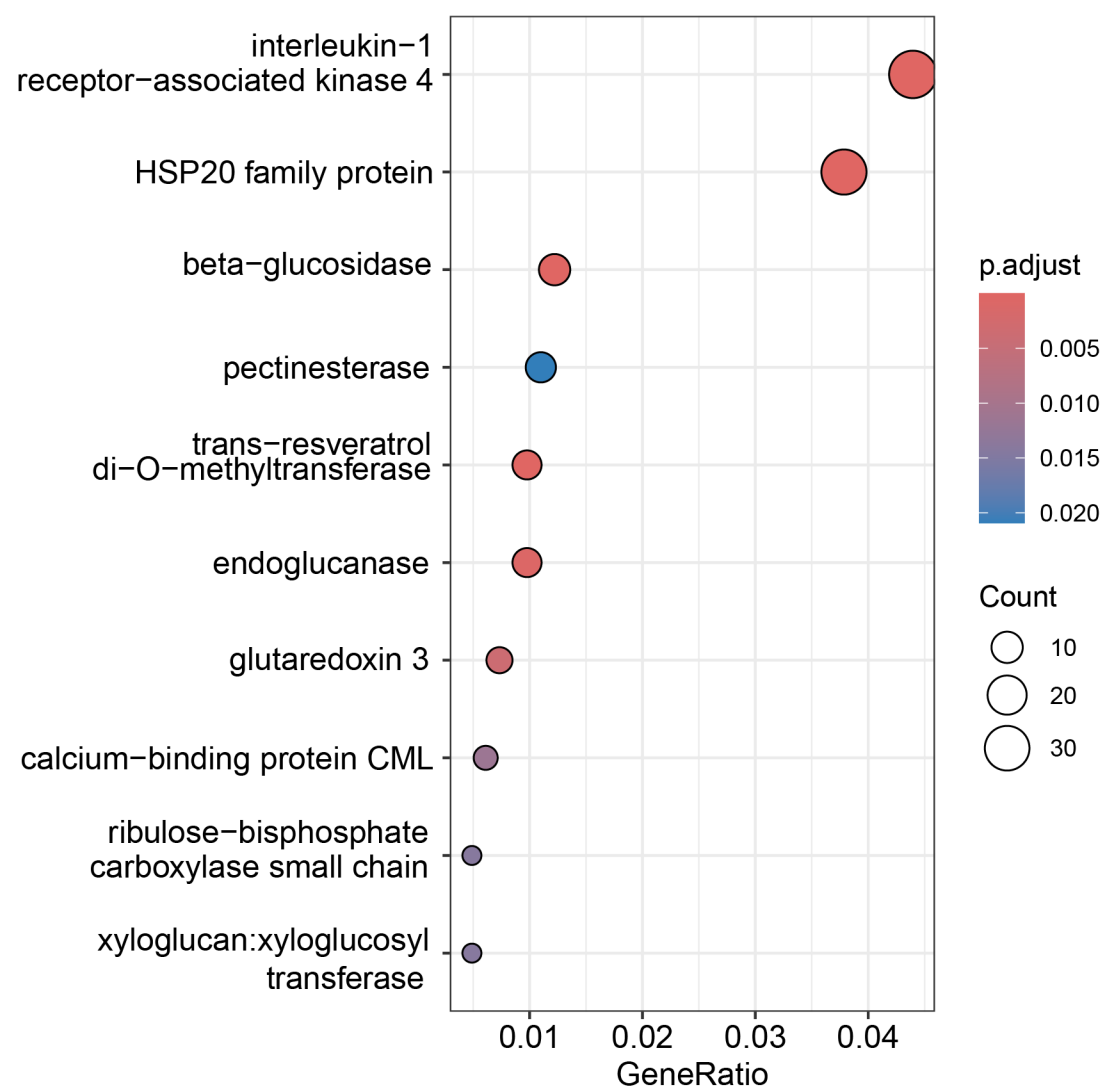

FOL

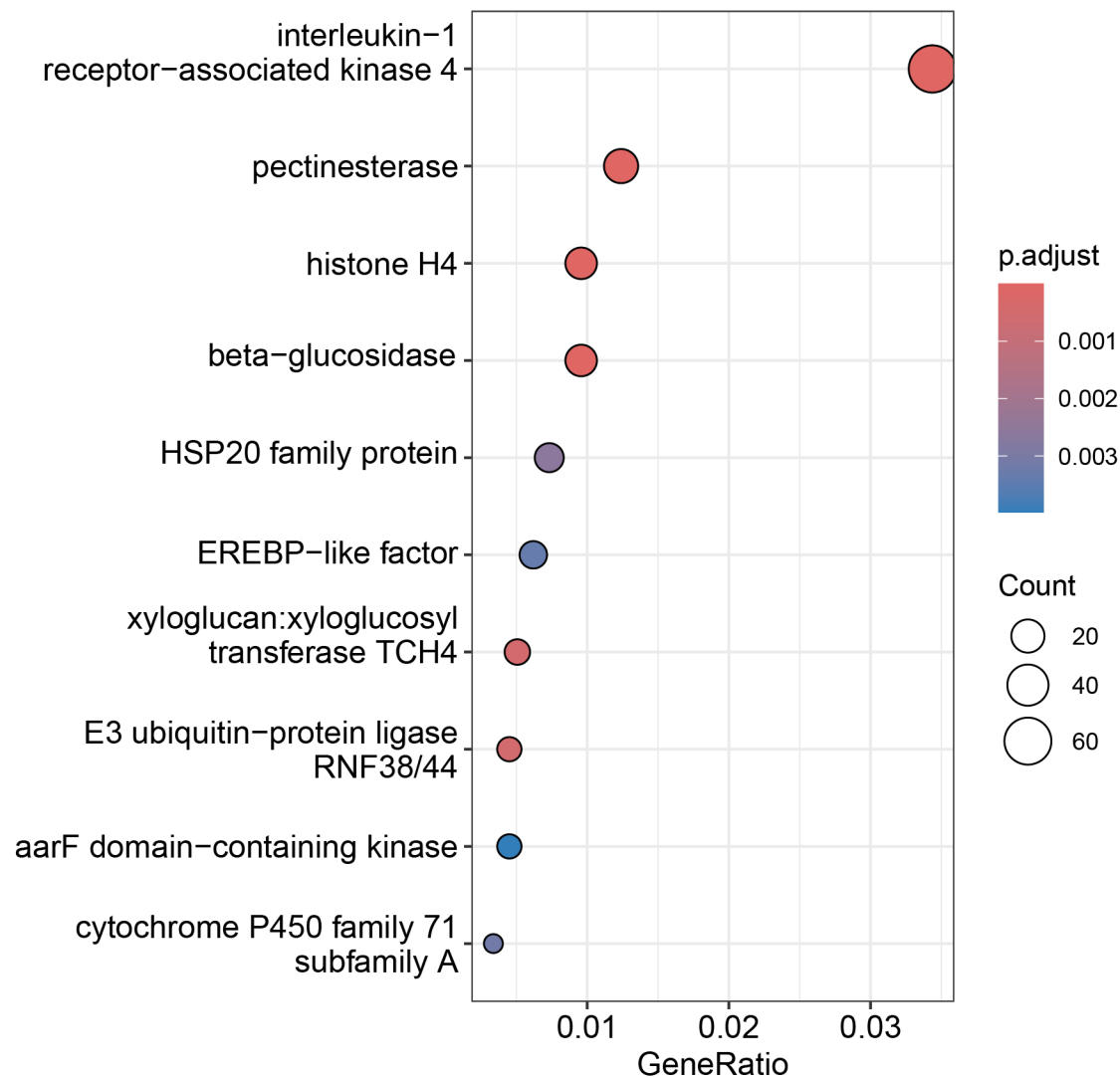

FS1

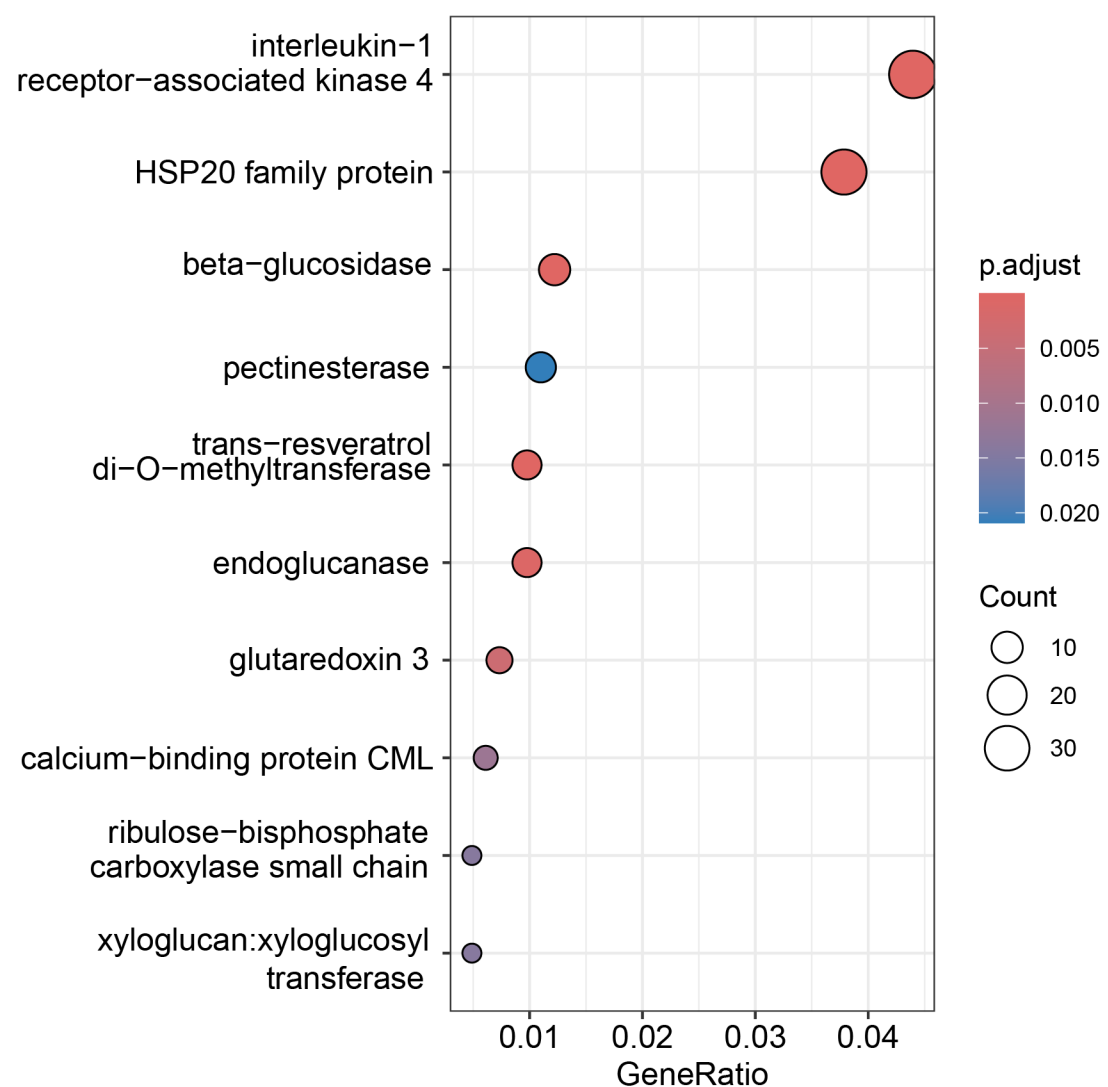

Supplement: Web_Material_uhaf006 [file web_material_uhaf006.zip › Figure S15.pdf]

A

2D PCA Plot

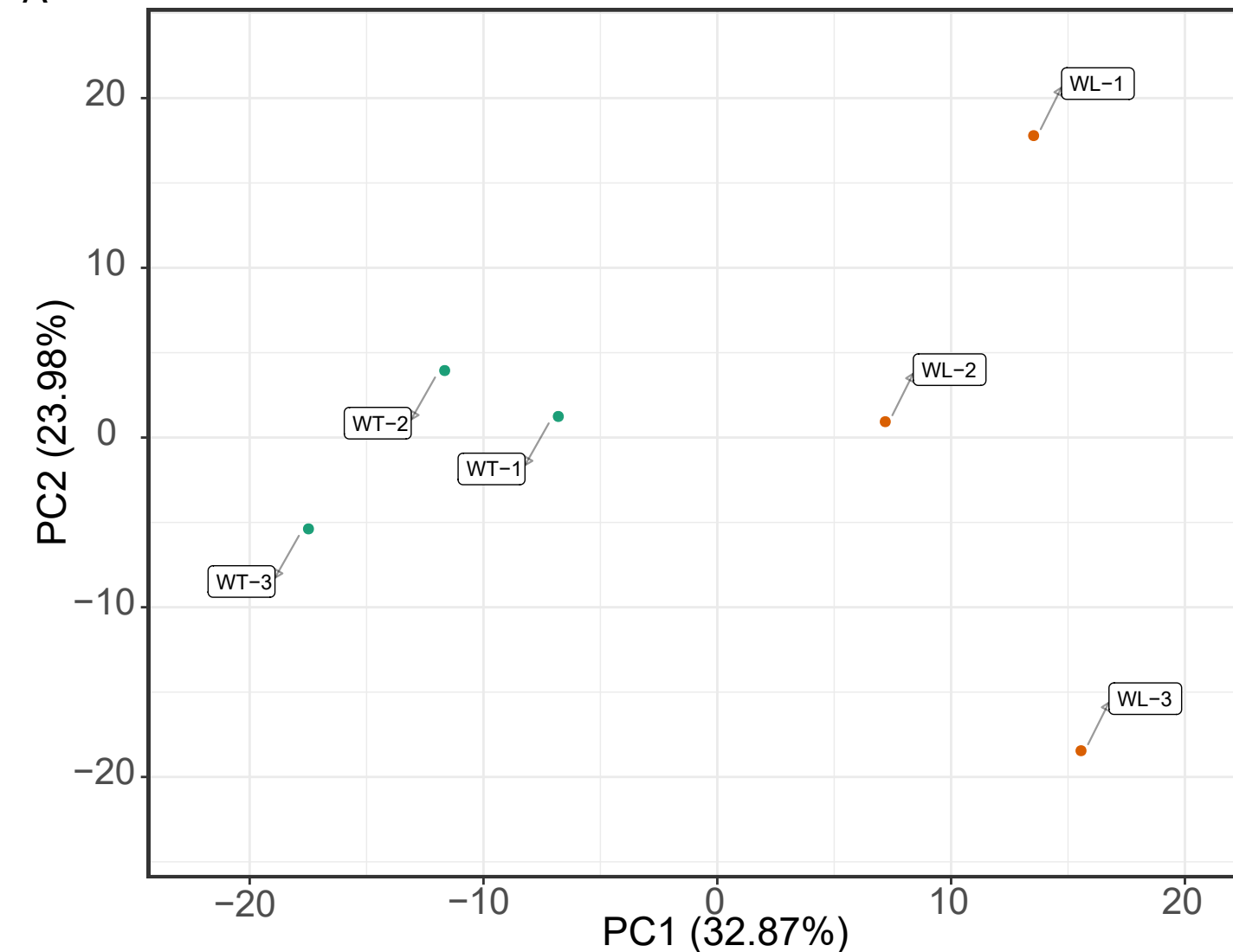

B

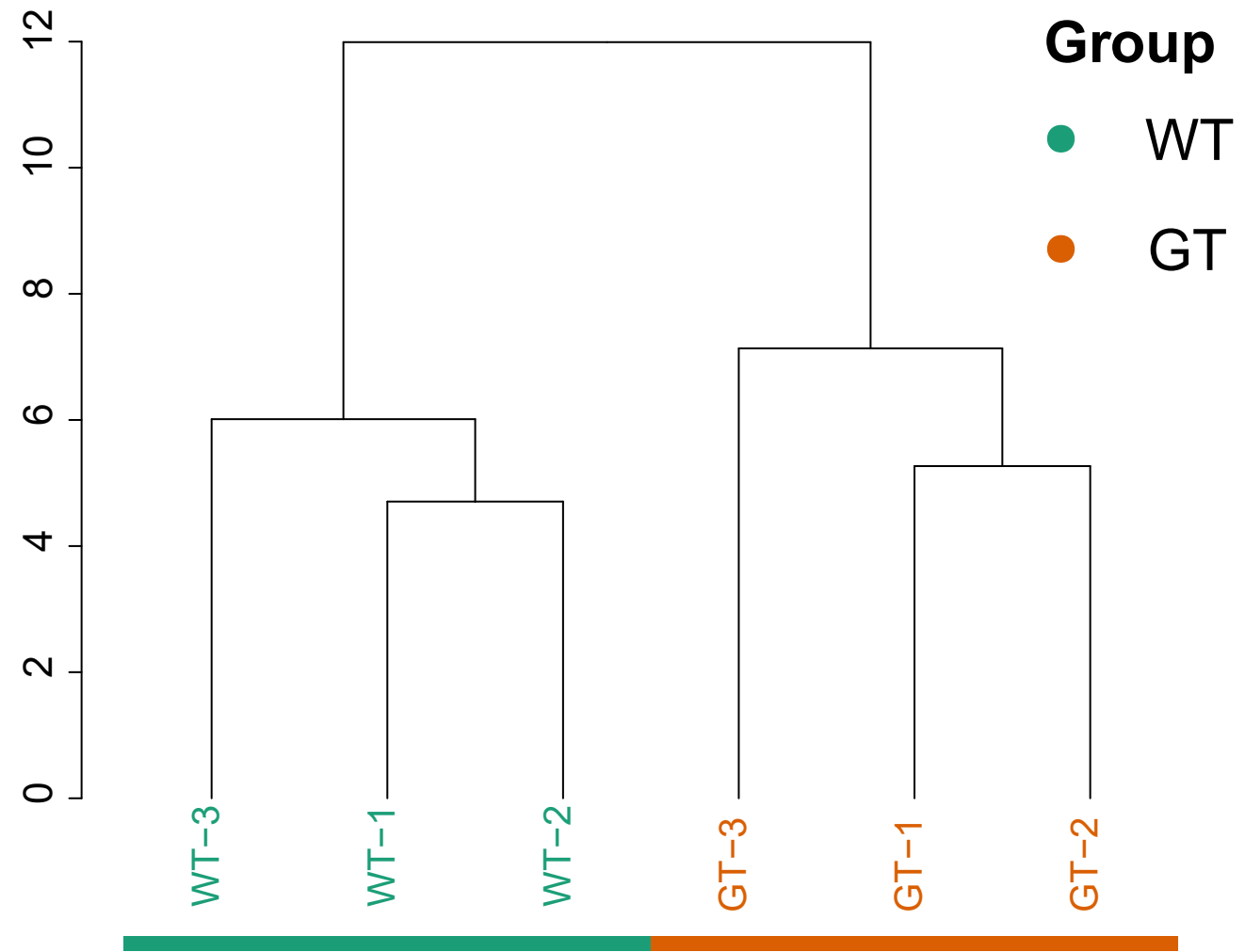

Supplement: Web_Material_uhaf006 [file web_material_uhaf006.zip › Figure S16.pdf]

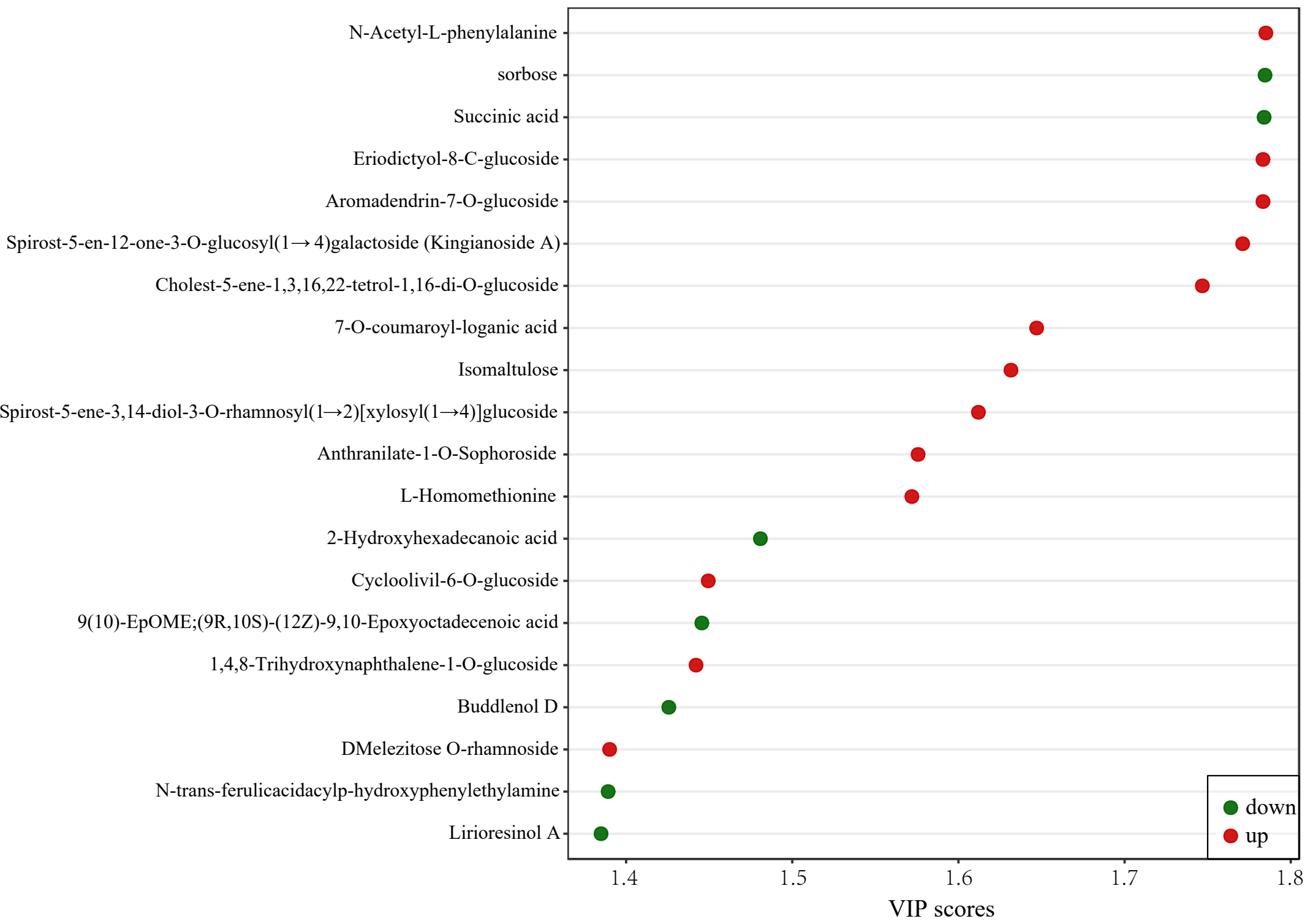

Supplement: Web_Material_uhaf006 [file web_material_uhaf006.zip › Figure S17.pdf]
