# Supplementary material for: A premature termination codon mutation in the onion AcCER2 gene is associated with both glossy leaves and thrip resistance
Source: Hortic Res. 2025 Jan 14;12(4):uhaf006. doi: 10.1093/hr/uhaf006 (PMC11896967; doi:10.1093/hr/uhaf006)
Supplement: Web_Material_uhaf006 [file Figure S3.pdf › Figure S3.pdf]

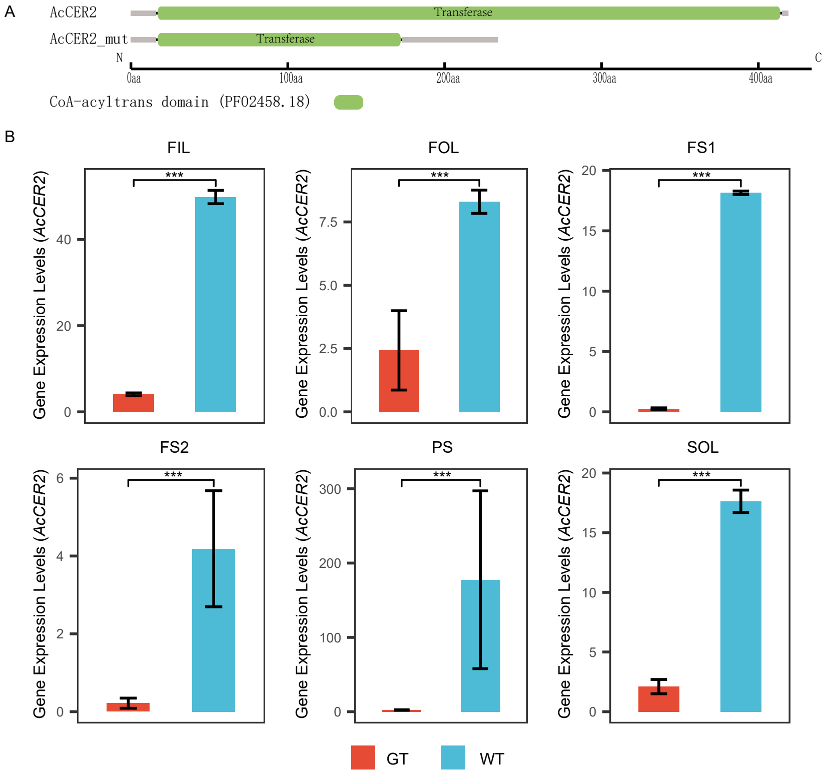

A
AcCER2
Transferase
AcCER2_mut
Transferase
N
C
0aa
100aa
200aa
300aa
400aa
CoA-acyltrans domain (PF02458.18)
B
FIL
FOL
FS1
FS2
PS
SOL
***
Gene Expression Levels (AcCER2)
GT
WT
